# ﻿Polyclads (Platyhelminthes) in the southern Gulf of Mexico: unveiling biodiversity and descriptions of two new species

**DOI:** 10.3897/zookeys.1221.128260

**Published:** 2024-12-13

**Authors:** Daniel Cuadrado, Alejandro Hernández-Gonzalez, Carolina Noreña, Nuno Simões

**Affiliations:** 1 Department of Biodiversity and Evolutionary Biology, National Museum of Natural Science, MNCN (CSIC), Madrid 28006, Spain National Museum of Natural Science, MNCN (CSIC) Madrid Spain; 2 Universidad Nacional Autónoma de México, Merida, Mexico Universidad Nacional Autónoma de México Merida Mexico; 3 Laboratorio Nacional de Resiliencia Costera, México, Mexico Laboratorio Nacional de Resiliencia Costera México Mexico; 4 International Chair for Coastal and Marine Studies, Harte Research Institute for Gulf of Mexico Studies, Corpus Christi, USA Harte Research Institute for Gulf of Mexico Studies Corpus Christi United States of America

**Keywords:** Campeche, flatworms, histological analysis, marine invertebrates, new record, Quintana Roo, species discovery, taxonomy, Yucatan

## Abstract

The order Polycladida (Platyhelminthes) in Mexico has historically received limited attention from researchers, primarily due to challenges associated with its low detectability and the scarcity of specialists. This study addresses part of the gap by conducting a comprehensive assessment of polyclad diversity in the southern Gulf of Mexico. Our investigation revealed a total of 27 distinct species, belonging to 17 genera and 12 families, within the suborders Cotylea and Acotylea. Our findings include the identification of 17 species previously undocumented in the Gulf of Mexico. This represents a significant expansion of the region’s known polyclad biodiversity. By revising the polyclad records in the Gulf of Mexico, the known species count has increased from 31 to 50. Furthermore, our research unveiled the presence of two new species, *Stylochoplanasisalensis***sp. nov.** and *Emprosthopharynxhartei***sp. nov.**, also marking the first time a species of the genus *Emprosthopharynx* has been reported for the Atlantic coast of the Americas.

## ﻿Introduction

The Gulf of Mexico (GoM) is renowned for being one of the largest marine ecosystems worldwide, due to its unique combination of hydrographic factors, biological productivity, and population diversity. Covering more than 1.5 million km^2^, the GoM boasts a broad range of marine habitats, from coral reefs to fishing banks and coastal areas, making it a globally significant ecosystem (Chávez-Hidalgo 2009). Additionally, the interaction of ocean currents, winds, and unique geomorphological features contributes to the distinctive dynamics of this marine ecosystem. This optimal environment supports marine life and makes the GoM an essential area for the reproduction, feeding, and migration of numerous marine species (Gil-Agudelo et al. 2020).

Polyclads, marine flatworms, have not received much attention from researchers in Mexico. There are several reasons for the difficulty of observing these small organisms, including their ability to mimic their surroundings. More than 1000 species of polyclads are known worldwide according to Tyler et al. (2006–2024). Despite this, research on these organisms in GoM is limited. The largest number of records and species known in the Gulf of Mexico region was discovered in the initial studies conducted by Pearse and Wharton (1938) and by Hyman (1940, 1944, 1954) along the US coasts of Louisiana, Texas, and Florida. *Prostheceraeuscrozieri*, a species also studied in this work, was documented in Florida by Newman et al. (2000). Quiroga et al. (2004a, 2004b) identified 124 species in the Gulf of Mexico and the Caribbean, while 40 species were found in Florida and 26 species were distributed throughout the Gulf of Mexico (Hooge and Newman 2009). Lastly, Quiroga (2008) described two new polyclad species in Louisiana, which is the latest discovery to date. The study conducted by Rawlinson (2008) focused on the polyclad species of the Caribbean Sea, which included Florida.

**Figure 1.**
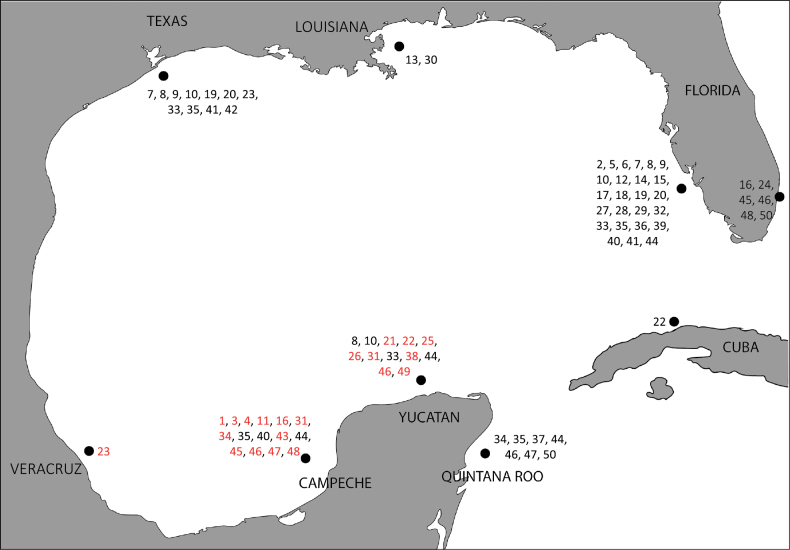
Records of the order Polycladida in the Gulf of Mexico as reported in Suppl. material 1: table S1. The numbers in red indicate new records for the Gulf of Mexico. 1. *Stylochoplanasisalensis* sp. nov., 2. *Phaenocelispurpurea*, 3. *Phaenocelismedvedica*, 4. *Phaenocelispeleca*, 5. *Coronadenamutabilis*, 6. *Spinantiapellucida*, 7. *Gnesiocerosfloridana*, 8. *Gnesiocerossargassicola*, 9. *Chatziplanagrubei*, 10. *Hoploplanainquilina*, 11. *Hoploplanadivae*, 12. *Latocestuswhartoni*, 13. *Didangiacarneyi*, 14. *Digynoporaamericana*, 15. *Euplanagracilis*, 16. *Notoplanaannula*, 17. *Comoplanaangusta*, 18. *Stylochusoculifera*, 19. *Stylochusellipticus*, 20. *Stylochusfrontalis*, 21. *Stylochussixteni*, 22. *Idioplanaatlantica*, 23. *Notocomplanaferruginea*, 24. *Notocomplanalapunda*, 25. *Emprosthopharynxhartei* sp. nov., 26. *Euryleptaaurantiaca*, 27. *Euryleptamulticelis*, 28. *Acerotisabaiae*, 29. *Oligocladusfloridanus*, 30. *Oligocladusbathymodiensis*, 31. *Prostheceraeuscrozieri*, 32. *Prostheceraeusfloridanus*, 33. *Pericelisorbicularia*, 34. *Periceliscata*, 35. *Enchiridiumperiommatum*, 36. *Prosthiostomumlobatum*, 37. *Prosthiostomumutarum*, 38. *Enchiridiumevelinae*, 39. *Acanthozoonmaculosum*, 40. *Thysanozoonbrocchii*, 41. *Thysanozoonnigrum*, 42. *Pseudoceros* (?) *texanus*, 43. *Pseudocerosjuani*, 44. *Pseudocerosbicolor*, 45. *Pseudocerosbolool*, 46. *Pseudocerosrawlinsonae*, 47. *Phrikocerosmopsus*, 48. *Pseudobiceroscaribbensis*, 49. *Pseudobicerossplendidus*, 50. *Pseudobicerospardalis*.

The present work is a significant contribution to the region as it addresses the shortage of species records and the urgent need to update the biodiversity inventory of the order Polycladida in the Gulf of Mexico (GoM). By examining 142 specimens, we identified 27 taxa that belong to 12 families and 17 genera, increasing the known species count from 31 to 50 (Suppl. material 1: table S1, Fig. 1). The Gulf documentation now includes 17 species that were not previously recorded and the discovery of two new species, which are described below.

## ﻿Materials and methods

### ﻿Sampling sites and processing of material

**Figure 2.**
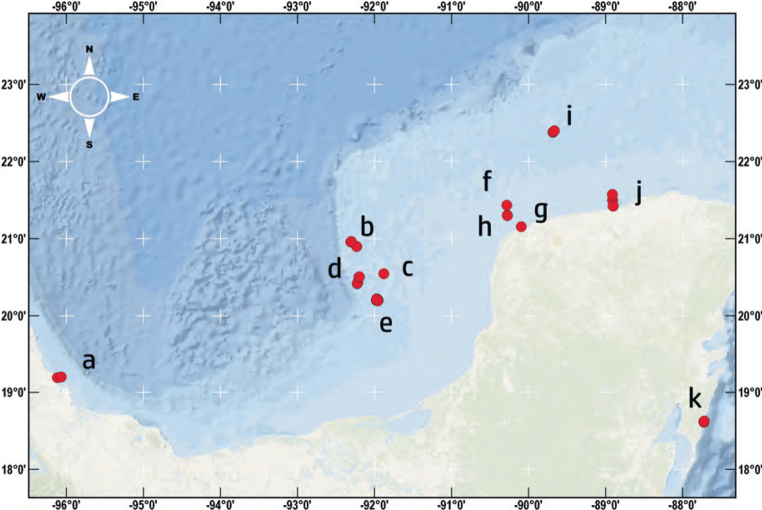
Sampling sites in the southern Gulf of Mexico and the Mexican Caribbean **a** Veracruz **b** West Triangles reef and East **c** reefs Banco Nuevo and Banco Pera **d** Banco Obispos north and south **e** Cayo Arcas **f** reef Madagascar **g** Punta Piedra **h** Bajo de Sisal **i** Alacranes Reef **j** Dzilam de Bravo **k** Mahahual.

The study material from the southern Gulf of Mexico was obtained through direct field collection using scuba diving and snorkelling in the subtidal (Fig. 2). Comprehensive information about external features was meticulously recorded using notes, photographs, and drawings. Details regarding pigmentation, colour patterns, movement, size, and the presence or absence of tentacles or eyes were documented and have been used for the species descriptions. Additionally, dorsal structures such as papillae, stripes, warts, or any type of epithelial or dermal formations were compiled. Photographs in the field in their habitat were taken whenever it was possible with a Canon G16. Photographs of the living specimens were taken to document their colouration. Whenever possible, the photographs were taken on a black background using transmitted light with a Nikon D90 camera equipped with a Micro Nikkor 60 mm lens.

### ﻿Histological processing

To ensure proper fixation, individuals were first anaesthetised with a seawater/magnesium chloride solution (7%). A small tissue sample was extracted and preserved in absolute ethanol for future molecular studies, and the entire specimen was then fixed in Bouin’s solution (saturated picric acid solution, formaldehyde, and acetic acid in a 15:5:1 proportion) for histological studies (Romeis 1989). After embedding in paraffin or paraplast, histological sagittal sections were cut, ranging from 6 to 12 micrometres in thickness, and subsequently stained using AZAN and Mallory trichrome, as well as hematoxylin-eosin staining techniques. For the definitive identification of genus/species, internal anatomical reconstructions, particularly of the reproductive apparatus, were performed using a Zeiss Axio Scope A1 microscope.

Whole body specimens and the histological preparations were deposited at the Colección regional de Policladidos de la Península del Yucatán, Mexico (**CRPPY**), located at the Unidad Multidisciplinaria de Docencia e Investigación de Sisal, Facultad de Ciencias, Universidad Nacional Autónoma de México (UMDI-Sisal, FC-UNAM).

### ﻿Abbreviations used in the figures

**cg**: cement glands, **co**: copulatory organ, **ct**: connective tissue, **de**: ejaculatory duct, **dlv**: Lang’s vesicle duct, **e**: stylet, **ed**: ejaculatory duct, **e-gpt**: epithelial-glandular prostate tissue, **ev**: external vagina, **fa**: female atrium, **fg**: female gonopore, **i**: intestine, **iv**: internal vagina, **lv**: Lang’s vesicle, **m**: muscle layer, **ma**: male atrium, **mb**: marginal band, **mg**: male gonopore, **ml**: marginal line, **oc**: cerebral ocelli/eyes, **om**: marginal ocelli/eyes, **ot**: tentacular ocelli/eyes, **ov**: oviduct, **p**: pharynx, **pa**: papillae, **pp**: penis papilla, **pt**: pseudotentacles, **pte**: pseudotentacle eyes, **pv**: prostatic vesicle, **s**: sucker, **sg**: shell glands, **sv**: seminal vesicle, **t**: tentacle, **va**: vagina, **vd**: vas deferens.

### ﻿DNA extraction and amplification

Total genomic DNA was extracted from each sample following the phenol-chloroform protocol (Chen et al. 2010). DNA concentration and purity of the extraction were measured using a NanoDrop Fluorospectrometer (Thermo Fisher Scientific). Sequences of the ribosomal gene 28S of the investigated Polycladida species were studied. All PCRs were performed using Taq DNA polymerase of Mastermix (Invitrogen, Carlsbad, CA) following the manufacturer’s protocol in a total volume of 25 μl. Sequences of approximately 1100 bp of the 28S gene were amplified with degenerated primers designed by Cuadrado et al. (2021): forward primer (5´-AGCCCAGCACCGAATCCT3-´) and reverse (5´-GCAAACCAAGTAGGGTGTCGC-3´). The PCR consisted of an initial denaturation step at 95 °C (3 min), followed by a pre-cycle of 5 cycles of denaturation at 96 °C (30 sec), annealing at 55 °C (30 sec) and extension at 72 °C (1 min), followed by 40 cycles of denaturation at 95 °C (30 sec), annealing at 59 °C (30 sec) and extension at 72 °C (1 min), with a final extension of 10 min at 72 °C.

The PCR products were observed using TBE gel electrophoresis in 1.5% agarose gel stained with SYBER Safe and visualised under UV light. PCR products were sent to Macrogen for clean-up and sequencing. Lastly, obtained forward and reverse sequences were combined using the program Geneious Prime v. 2020.2.4 (http://www.geneious.com, Kearse et al. 2012) using the alignment-transition/transversion with the consensus sequence tool and manually created.

All sequences obtained in the present study have been deposited in the GenBank database under the accession numbers included in Suppl. material 1: table S2.

## ﻿Results

### ﻿Polycladida

**Suborder Cotylea**

**Periceloidea Bahia, Padula & Schrödl, 2017**

**Pericelidae Laidlaw, 1902**

***Pericelis* Laidlaw, 1902**

#### ﻿ Pericelis cata

Taxon classificationAnimalia﻿PolycladidaPericelidae

Marcus & Marcus, 1968

D9EF86F3-F99A-5959-89AE-3E9F64631225

Fig. 3

##### Material examined.

**Campeche coast, Mexico** • 1; Cayos sumergidos del Oeste; 20.9°N, 92.2°W; 0 m; 10 Sep. 2017; A. Gutiérrez leg.; CRPPY-0011 • 1; Cayos sumergidos del Oeste; 20.9°N, 92.2°W; 13 m; 14 Sep. 2017; A. Gutiérrez leg.; CRPPY-0020 • 1; Cayos sumergidos del Oeste; 21.0°N, 92.3°W; 10 m; 9 Sep. 2017; F. Márquez leg.; CRPPY-0022 • 2; Cayos sumergidos del Oeste; 20.4°N, 92.2°W; 10.8 m; 14 Sep. 2017; A. Gutiérrez leg.; CRPPY-0024 • 1; Cayos sumergidos del Oeste; 20.5°N, 92.2°W; 26 m; 13 Sep. 2017; X. Vital leg.; CRPPY-0025; **Quintana Roo coast, Mexico** • 1; Mahahual; 18.6°N, 87.7°W; 13.4 m; 18 Mar. 2018; A. Hernández leg.; CRPPY-0040; **Campeche coast, Mexico** • 1; Cayo Arcas; 20.2°N, 92.0°W; 5 m; 19 Apr. 2018; A. Hernández leg.; CRPPY-0046 • 1; Cayo Arcas; 20.2°N, 92.0°W; 4.7 m; 19 Apr. 2018; A. Hernández leg.; CRPPY-0051 • 1; Cayo Arcas; 20.2°N, 92.0°W; 6.3 m; 22 Apr. 2018; A. Hernández leg.; CRPPY-0065 • 1; Cayo Arcas; 20.2°N, 92.0°W; 3.2 m; 24 Apr. 2018; A. Hernández leg.; CRPPY-0078 • 1; Cayo Arcas; 20.2°N, 92.0°W; 7.7 m; 25 Apr. 2018; A. Hernández leg.; CRPPY-0083.

##### Distribution.

This species was previously recorded in Curaçao (Marcus and Marcus 1968); the Caribbean coast of Colombia (Quiroga et al. 2004a, 2004b); Cabo Frío, Salvador, and Alagoas, Brazil (Queiroz et al. 2013; Bahia et al. 2014, 2015; Bahia and Schrödl 2018); Canary Islands, Spain (Cuadrado et al. 2017). This is a new record for the coasts of Campeche (Gulf of Mexico), and Quintana Roo (Mexican Caribbean). New record for the Gulf of Mexico.

##### Description.

**Figure 3.**
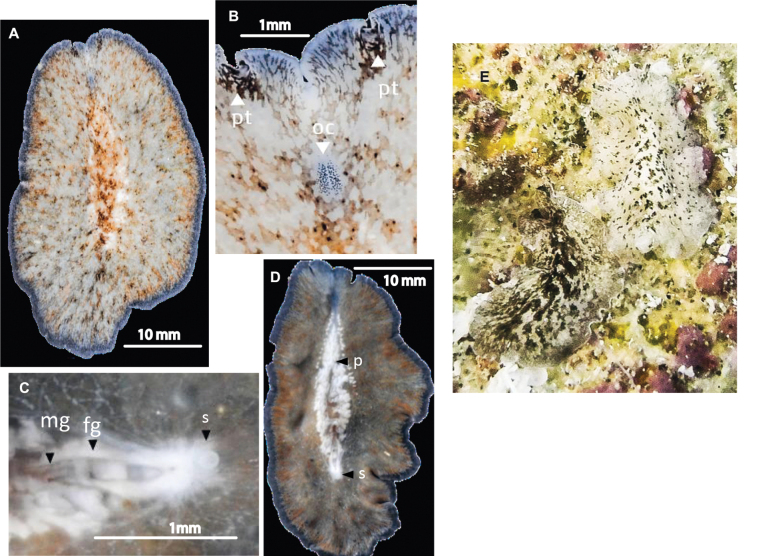
*Periceliscata***A** dorsal view **B** frontal region, cerebral eyes and pseudotentacles **C** location of the sucker, male and female gonopores **D** ventral view **E***P.cata* in situ.

Body oval with multiple marginal folds, 4 cm in length and 2.5 cm in width. Dorsally, pattern of dark pigmentation is interrupted by spots where the white parenchyma is observed. Towards the margin, the white patches become smaller, and the space between them decreases, with scattered black dots. The tentacles are subtle marginal folds, with a clear separation between them, characteristic of the genus *Pericelis* (Fig. 3A, B). Marginal eyes are arranged irregularly around the entire body margin (Fig. 3A, B). Dorsally, the tentacular and cerebral eyes are arranged in two elongated clusters (Fig. 3B).

##### Remarks.

*Periceliscata* morphology found in the Gulf of Mexico corresponds to the original description by Marcus and Marcus (1968), characterised by the position of the pseudotentacles, the elongated cerebral eye clusters, and the colour pattern: white and black spots on a brown background (Fig. 3A−D).The pigmentation of the specimens sampled in the Gulf of Mexico is different from that described in the original description of *P.cata*. The Mexican species displays a basal colour of white, with brown spots and freckles (as seen in Fig. 3E).

#### ﻿ Pericelis orbicularia

Taxon classificationAnimalia﻿PolycladidaPericelidae

(Schmarda, 1859)

BE5A3627-BA91-5CC3-B501-D863FA1A911A

Fig. 4

##### Material examined.

**Yucatan coast, Mexico** • 1; Punta Piedra, Sisal; 21.2°N, 90.1°W; 1 m; 30 Apr. 2018; A. Hernández leg.; CRPPY-0087 • 1; Dzilam; 21.5°N, 88.9°W; 9.3 m; 8 May 2018; A. Hernández leg.; CRPPY-0091 • 1; 12 slides; Dzilam; 21.5°N, 88.9°W; 13 m; 10 May 2018; A. Hernández leg.; CRPPY-0097.

##### Distribution.

*Pericelisorbicularia* is known from the south coast of Jamaica (Schmarda 1859); Port Aransas, Texas, USA (Hyman 1955); and Key Biscayne (Florida, USA; Marcus and Marcus 1968). This is the first record for the coast of Yucatan (Mexico).

##### Description.

**Figure 4.**
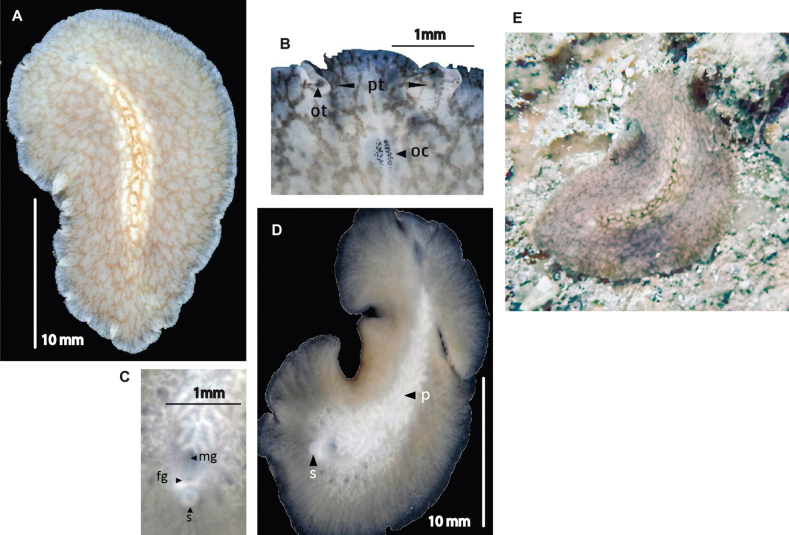
*Pericelisorbicularia***A** dorsal view **B** shape of pseudotentacles, pseudotentacular eyes, cerebral eyes **C** male and female gonopores, sucker **D** ventral view **E***P.orbicularia* in situ.

Body oval-shaped, 2 cm in length and 1 cm in width, with small pseudotentacles, less than 1 mm. Dorsal surface exhibits an orange to light brown reticulated pattern on a regular creamy beige background (Fig. 4A, D, E). The pigmentation corresponds to the colouration described by Hyman (1955) for specimens of Port Aransas, Texas. According to Hyman, *Pericelisorbicularia* presents “a reddish-brown network on a paler ground”. Cerebral eyes are arranged in two elongated clusters. Tentacular and marginal eyes scattered along the margin. A swelling in the body’s midline is caused by the highly folded pharynx and the copulatory organ (Fig. 4A, B, E). *Pericelisorbicularia* was observed to secrete an abundant and viscous mucus. ***Reproductive system***. The male and female copulatory apparatus are located just posterior to the pharynx and before the prominent sucker, 0.5 mm distance between them. Live specimens exhibit distinct female and male gonopores, but in our fixed specimens, the gonopores appear as a concavity, giving the impression of a single gonopore (Fig. 4C, D). The male copulatory apparatus shows an anteroposterior orientation, including a highly muscular seminal vesicle and an ejaculatory duct lined with glandular epithelium.

### ﻿Prosthiostomidae Lang, 1884

***Prosthiostomum* Quatrefage, 1845**

#### ﻿ Prosthiostomum utarum

Taxon classificationAnimalia﻿Polycladida﻿Prosthiostomidae

Marcus, 1952

90B7A43C-C12D-5008-AB5B-9BFEFBDF823A

Fig. 5A

##### Material examined.

**Quintana Roo coast, Mexico** • 1; Mahahual; 18.6°N, 87.7°W; 13.4 m; 18 Mar. 2018; A. Hernández leg.; CRPPY-0041.

##### Distribution.

This species was described from Sao Sebastiao Island, Brazil (type locality; Marcus 1952) and the Praia das Conchas, Cabo Frío, Brazil (Bahia et al. 2014; Bahia and Schrödl 2018), as well as on the Atlantic coast of Florida. It has also been recorded in the Caribbean Sea, Colombia (Quiroga et al. 2004a, 2004b). The discovery of *Prosthiostomumutarum* on the coast of Quintana Roo presents a new record for this species in the Mexican Caribbean Sea.

##### Description.

**Figure 5.**
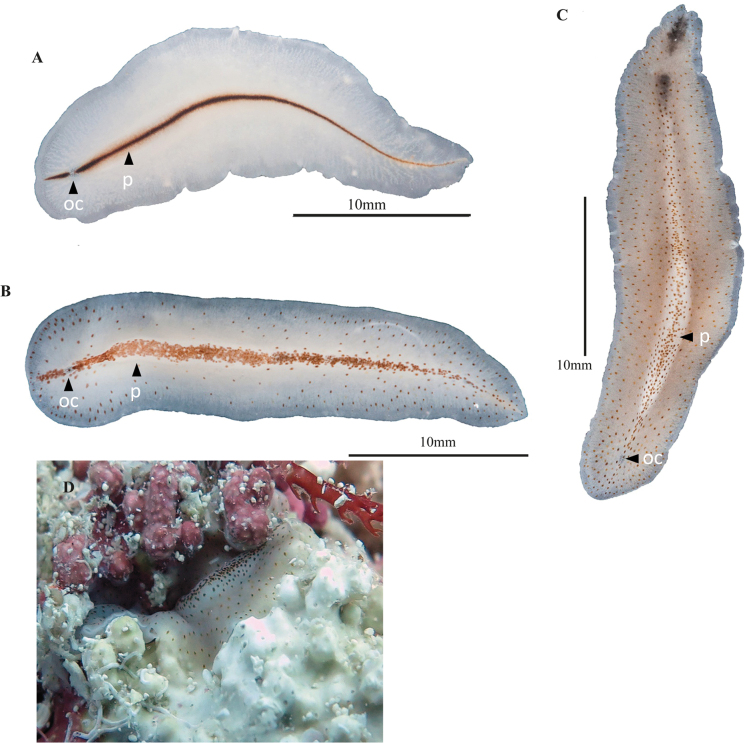
**A***Prosthiostomumutarum* dorsal view **B***Enchiridiumperiommatum* in situ **C***Enchiridiumevelinae* dorsal view **D***Enchiridiumevelinae* in situ.

Body shape elongated, 3 cm in length and 0.7 cm in width with a rounded anterior end and a pointed posterior end (Fig. 5A). Predominantly white tonalities and distinctive brown pigmentation along the midline and the anterior region. Cerebral eyes organised in two elongated clusters. Marginal eyes along the anterior region.

##### Remarks.

*Prosthiostomumutarum*, originally described as *Lurymareutarum* (Marcus, 1952), was recently reassigned to the genus *Prosthiostomum* based on the work of Litvaitis et al. (2019) based on the 28S gene. The morphology of Gulf of Mexico specimens corresponds to the original description by Marcus (1952). A comprehensive investigation, including both morphological and molecular aspects, is necessary for accurate delimitation of the genera *Lurymare* and *Prosthiostomum*.

### ﻿*Enchiridium* Bock, 1913

#### ﻿ Enchiridium evelinae

Taxon classificationAnimalia﻿Polycladida﻿Prosthiostomidae

Marcus, 1949

D72C62E3-C29F-538D-8552-5E7BD6A0CD23

Fig. 5C, D

##### Material examined.

**Yucatan coast, Mexico** • 1; Bajos de Sisal; 21.2°N, 90.0°W; 1 m; 10 Sep. 2017; A. Hernández leg.; CRPPY-0033.

##### Distribution.

Recorded in São Paulo (Marcus 1950), Rio Grande do Norte, and Alagoas (Bahia et al. 2012, 2014, 2015; Bahia and Schrödl 2018) in Brazil and Panama (Rawlinson 2008). It is also known in Curaçao (Marcus and Marcus 1968). This work represents a new record for the Yucatan coast. New record for the Gulf of Mexico.

##### Description.

Body shape elongated, 3 cm in length and 1 cm in width. Body cream-coloured with brown, orange, and yellow spots arranged densely along the midline and paler towards the margins (Fig. 5C, D). Tubular pharynx extends to ~ 1/3 of the body’s length. Reproductive male apparatus with an enclosed seminal vesicle and two prostatic vesicles included in a common muscular bulb, and a long penis papilla armed with a stylet opening in a long male atrium.

##### Remarks.

The spots disappear after fixation and, according to Marcus (1950), the pigmentation of these spots is lipoid (Fig. 5C, D). The specimens recorded here have a lower density of dots compared to the specimens described in Bahia et al. (2014: fig. 14).

#### ﻿ Enchiridium periommatum

Taxon classificationAnimalia﻿Polycladida﻿Prosthiostomidae

Bock, 1913

40B9D9D6-5855-548E-AB81-16EE69603B8F

Fig. 5B

##### Material examined.

**Yucatan coast, Mexico** • 1; Arrecife Alacranes; 22.4°N, 89.7°W; 1 m; 3 Nov. 2017; A. Hernández leg.; CRPPY-0001 • 1; Arrecife Alacranes; 22.4°N, 89.7°W; 1 m; 3 Nov. 2017; A. Hernández leg.; CRPPY-0003 • 2; Arrecife Alacranes; 22.4°N, 89.7°W; 1 m; 4 Nov. 2017; A. Hernández leg.; CRPPY-0005 • 1; Arrecife Alacranes; 22.4°N, 89.7°W; 2 m; 4 Nov. 2017; A. Hernández leg.; CRPPY-0007; **Quintana Roo coast, Mexico** • 1; Mahahual; 18.6°N, 87.7°W; 15 m; 18 Mar. 2018; A. Hernández leg.; CRPPY-0042; **Yucatan coast, Mexico** • 1; Dzilam; 21.5°N, 88.9°W; 13 m; 10 May 2018; A. Hernández leg.; CRPPY-0098.

##### Distribution.

The species was originally described in Thatch Island, US Virgin Islands (Bock 1913), later collected in Jamaica (Hyman 1955), and also known from Texas to Florida, Gulf of Mexico (Hyman 1955). This is a new record for the coasts of Campeche and Quintana Roo (Mexico).

##### Description.

Body elongated, 1.5 cm in length and 0.5 cm in width, with a rounded anterior end and a tapered posterior end. Marginal eyes densely distributed along the anterior margin; cerebral eyes in a heart-shaped cluster. Translucent white background speckled with dense brown to orange spots that gradually decrease in number towards the edges (Fig. 5B). Pharynx, male and female reproductive organs, as well as the sucker are located in the anterior 1/2 of the body, a distinctive feature of this species.

### ﻿Pseudocerotoidea Faubel, 1984

**Euryleptidae Stimpson, 1857**

***Eurylepta* Ehrenberg, 1831**

#### ﻿ Eurylepta aurantiaca

Taxon classificationAnimalia﻿PolycladidaEuryleptidae

Heath & McGregor, 1912

5F8173C8-9D22-5F5C-B221-7978F889E9AA

Fig. 6

##### Material examined.

**Yucatan coast, Mexico** • 1; Dzilam; 21.5°N, 88.9°W; 9.3 m; 8 May 2018; A. Hernández leg.; CRPPY-0088.

##### Distribution.

The species was recorded in Monterey Bay, California (Heath and McGregor 1912); Washington State, USA (Hyman 1955), the Caribbean Sea of Colombia (Quiroga et al. 2004a, 2004b); Brazil (Bahia et al. 2014); India (Pitale and Apte 2019). This work represents a new record for the Yucatan coast. New record for the Gulf of Mexico.

##### Description.

**Figure 6.**
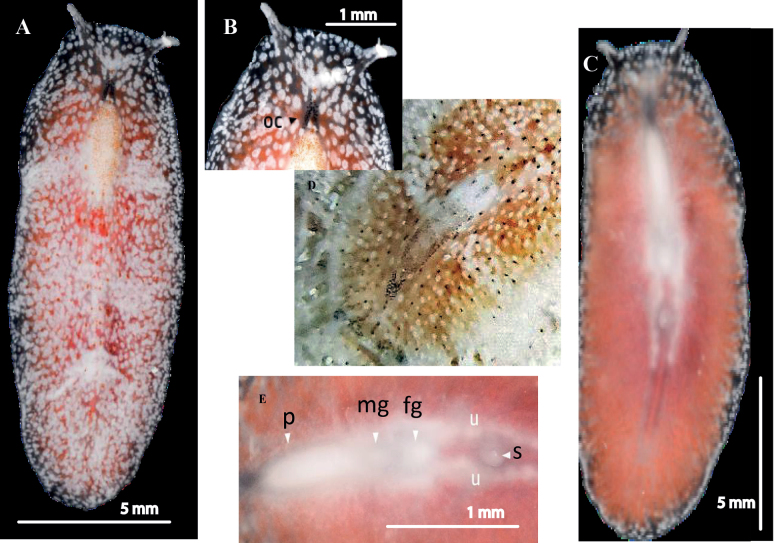
*Euryleptaaurantiaca***A** dorsal view **B** anterior region, cerebral eyes **C** ventral view **D***Euryleptaaurantiaca* in situ **E** detail of pharynx, male and female gonopores, uteri, and sucker.

Body shape elliptical, 1.7 cm in length and 0.5 cm in width, with a translucent to orange-pink colouration, white spots on the dorsal side, and a reddish median line with reddish dots (Fig. 6A, B, D). Intestinal branches apparent (Fig. 6C, E). Whitish tentacles. Cerebral eyes are in two elongated clusters, and tentacular eyes in the basal region of the tentacles. Two frontal eye clusters located between the tentacles.

##### Remarks.

The specimen collected from the Mexican coast exhibits a pigmentation characterised by a pinkish orange hue, as illustrated in Fig. 6A, congruent with the characterisation reported by Bahia et al. (2014).

### ﻿*Prostheceraeus* Schmarda, 1859

#### ﻿ Prostheceraeus crozieri

Taxon classificationAnimalia﻿PolycladidaEuryleptidae

(Hyman, 1939)

3E317940-9072-5152-8FF6-7764C4B2C4E9

Fig. 7

##### Material examined.

**Campeche coast, Mexico** • 1; Cayos sumergidos del Oeste; 20.4°N, 92.2°W; 18 m; 14 Sep. 2017; A. Gutiérrez leg.; CRPPY-0018 • 1; Cayo Arcas; 20.2°N, 92.0°W; 16.3 m; 20 Aug. 2018; A. Hernández leg.; CRPPY-0108.

##### Distribution.

Recorded in the east coast of Florida and the Florida Keys, Bermuda (Crozier 1917; Hyman 1939); Curaçao (Marcus and Marcus 1968); Jamaica, the Gulf of Mexico, and the Caribbean (Hyman 1952). New record for the coast of Campeche, Mexico.

##### Description.

**Figure 7.**
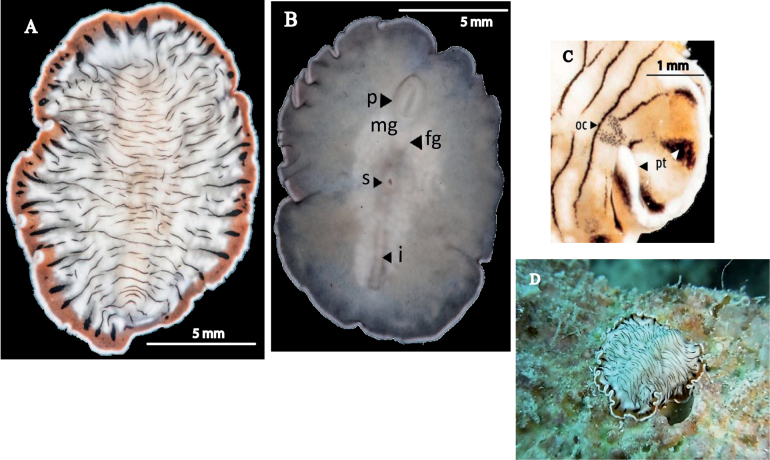
*Prostheceraeuscrozieri***A** dorsal view **B** ventral view. Detail of mouth and pharynx, female gonopore, sucker, intestine **C** cerebral eyes and pseudotentacles **E***P.crozieri* in situ.

Oval or circular-shaped body, 2.3 cm in length and 0.8 cm in width, with a semi-transparent white-beige background with transverse wavy black lines. The lines alternately end in a black spot or an orange blotch. Dorsal surface with white spots and a submarginal semi-transparent and marginal narrow white band (Fig. 7A, C, D). Ventral surface creamy white (Fig. 7B). Marginal orange tentacles long with black and white tips. Cerebral eye is distributed in two elongated clusters, each containing ~ 35 eyes (Fig. 7A, C). The anatomy of the reproductive system agrees with that described by Hyman (1939).

##### Remarks.

The specimen from the Gulf of Mexico aligns with the description of *Prostheceraeuscrozieri* provided by Hyman (1939). Newman et al. (2000) transferred both *Pseudoceroscrozieri* Hyman, 1939 and *Prostheceraeuszebra* Hyman, 1955 to *Maritigrellacrozieri* due to the presence of a tubular pharynx and the lack of uterine vesicles. Litvaitis et al. (2019) reclassified *Maritigrellacrozieri* as *Prostheceraeus* based on the description and illustration of *Prostheceraeuszebra* by Hyman (1955).

##### Biology.

*Prostheceraeuscrozieri* is documented as a primary consumer of the sea squirt *Ecteinascidiaturbinata*. Although this ascidian species was prolific within the research areas, the occurrence of *Prostheceraeuscrozieri* was limited.

### ﻿Pseudocerotidae Lang, 1884

***Pseudoceros* Lang, 1884**

#### ﻿ Pseudoceros bicolor

Taxon classificationAnimalia﻿Polycladida﻿Pseudocerotidae

Verrill, 1901

25BFD0B9-2E3F-52C9-BFEF-9FB0A2CC3DAD

Fig. 8

##### Material examined.

**Yucatan coast, Mexico** • 1; Arrecife Alacranes; 22.4°N, 89.7°W; 3 m; 4 Nov. 2017; A. Hernández leg.; CRPPY-0008; **Campeche coast, Mexico** • 1; Cayos sumergidos del Oeste; 20.4°N, 92.2°W; 10 m; 14 Sep. 2017; A. Hernández leg.; CRPPY-0015 • 1; Cayos sumergidos del Oeste; 20.4°N, 92.2°W; 11.1 m; 13 Sep. 2017; D. Ortigosa leg.; CRPPY-0027 • 1; Cayos sumergidos del Oeste; 20.4°N, 92.2°W; 11.7 m; 14 Sep. 2017; D. Ortigosa leg.; CRPPY-0028; **Yucatan coast, Mexico** • 1; Bajos de Sisal; 21.2°N, 90.0°W; 1 m; 22 Feb. 2018; A. Hernández leg.; CRPPY-0032; **Quintana Roo coast, Mexico** • 1; Mahahual; 18.6°N, 87.7°W; 7.7 m; 17 Mar. 2018; A. Hernández leg.; CRPPY-0039; **Campeche coast, Mexico** • 1; Cayo Arcas; 20.2°N, 92.0°W; 6.4 m; 19 Apr. 2018; A. Hernández leg.; CRPPY-0048 • 1; Cayo Arcas; 20.2°N, 92.0°W; 2.2 m; 20 Apr. 2018; A. Hernández leg.; CRPPY-0052 • 2; Cayo Arcas; 20.2°N, 92.0°W; 2.2 m; 20 Apr. 2018; A. Hernández leg.; CRPPY-0054 • 1; Cayo Arcas; 20.2°N, 92.0°W; 2.2 m; 20 Apr. 2018; A. Hernández leg.; CRPPY-0055 • 1; Cayo Arcas; 20.2°N, 92.0°W; 2.2 m; 21 Apr. 2018; A. Hernández leg.; CRPPY-0061 • 1; Cayo Arcas; 20.2°N, 92.0°W; 2.2 m; 22 Apr. 2018; A. Hernández leg.; CRPPY-0063 • 2; Cayo Arcas; 20.2°N, 92.0°W; 6.3 m; 22 Apr. 2018; A. Hernández leg.; CRPPY-0067 • 1; Cayo Arcas; 20.2°N, 92.0°W; 7.5 m; 23 Apr. 2018; A. Hernández leg.; CRPPY-0069 • 1; Cayo Arcas; 20.2°N, 92.0°W; 5.9 m; 23 Apr. 2018; A. Hernández leg.; CRPPY-0070 • 1; Cayo Arcas; 20.2°N, 92.0°W; 5.3 m; 23 Apr. 2018; A. Hernández leg.; CRPPY-0072 • 1; Cayo Arcas; 20.2°N, 92.0°W; 5.3 m; 24 Apr. 2018; A. Hernández leg.; CRPPY-0074 • 1; Cayo Arcas; 20.2°N, 92.0°W; 9.9 m; 25 Apr. 2018; A. Hernández leg.; CRPPY-0081 • 1; Cayo Arcas; 20.2°N, 92.0°W; 7.7 m; 25 Apr. 2018; A. Hernández leg.; CRPPY-0084 • 1; Cayo Arcas; 20.2°N, 92.0°W; 3.4 m; 18 Aug. 2018; A. Hernández leg.; CRPPY-0106 • 1; Cayo Arcas; 20.2°N, 92.0°W; 3.4 m; 18 Aug. 2018; A. Hernández leg.; CRPPY-0107.

##### Distribution.

Recorded in the Birds Islands, Bermuda (Verrill 1901); Curaçao (Marcus and Marcus 1968); the Caribbean coast of Colombia (Quiroga et al. 2004a); Florida, Virgin Islands, Jamaica, Belize, Honduras, Caribbean coast of Panama (Rawlinson 2008; Litvaitis et al. 2019); Brazil (Bahia and Padula 2009; Bahia et al. 2014, 2015; Bahia and Schrödl 2018). New record for the coasts of Campeche, Yucatán, and Quintana Roo (Mexican Caribbean), Mexico.

##### Description.

**Figure 8.**
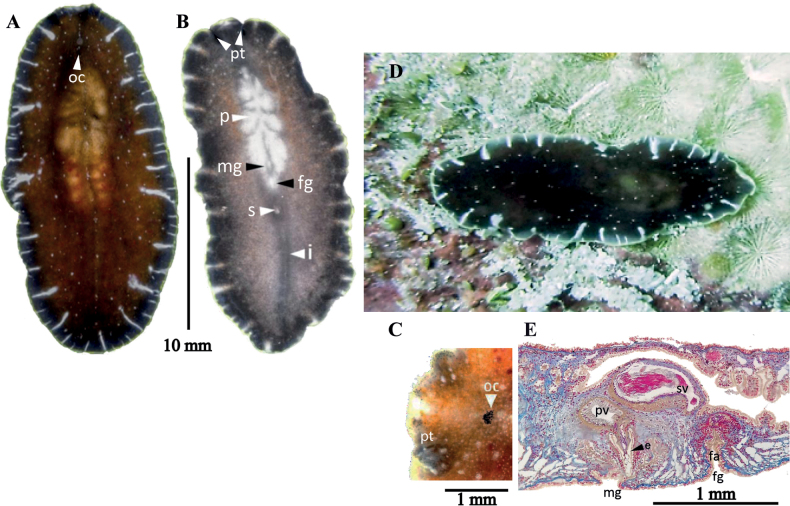
*Pseudocerosbicolor***A** dorsal view **B** ventral view, sucker, oral pore, pharynx, male gonopore, female gonopore and intestine **C** detail of tentacular eyes, pseudotentacles **D***P.bicolor* in situ **E** sagittal section, prostatic vesicle, seminal vesicle, male gonopore, stylet, vagina, female gonopore, female atrium Azan stained.

Body shape elongated with rounded anterior and posterior end, 2.5 cm in length and 1 cm in width. Dorsal pigmentation ranges from yellow to dark brown, with scattered white dots on its dorsal surface and with a yellowish or pale green marginal rim. Conspicuous dark marginal band interrupted by transverse white stripes (Fig. 8A, B, D). Pseudotentacles are simple folds with two clusters of eyes between them. Cerebral eyes arranged in the shape of an arrowhead, marginal eyes very numerous (Fig. 8C). Male and female gonopores located in the mid-region of the body, sucker posteriorly (Fig. 8B, E).

##### Remarks.

The pigmentation observed in *P.bicolor* in Yucatan aligns with the morphotype seen in Brazil (Litvaitis et al. 2010: fig. 4N). Preliminary analysis of the sequences obtained for the species (Suppl. material 1: table S2) suggests that Mexican specimens appear to have a closer genetic relationship to those in Brazil than those in the Caribbean Sea and nearby regions. A comparative molecular analysis will help to understand their genetic relationship with other morphotypes described in the literature.

#### ﻿ Pseudoceros rawlinsonae

Taxon classificationAnimalia﻿Polycladida﻿Pseudocerotidae

Bolaños, Quiroga & Litvaitis, 2007

90670214-AE3F-58BE-81F5-0A439CE270D9

Fig. 9

##### Material examined.

**Yucatan coast, Mexico** • 1; Bajos de Sisal; 21.2°N, 90.0°W; 1 m; 22 Feb. 2018; A. Hernández leg.; CRPPY-0029; **Quintana Roo coast, Mexico** • 1; Mahahual; 18.6°N, 87.7°W; 5.3 m; 17 Mar. 2018; A. Hernández leg.; CRPPY-0036; **Campeche coast, Mexico** • 1; Cayo Arcas; 20.2°N, 92.0°W; 9.3 m; 21 Apr. 2018; A. Hernández leg.; CRPPY-0056 • 1; Cayo Arcas; 20.2°N, 92.0°W; 5 m; 25 Apr. 2018; A. Hernández leg.; CRPPY-0085.

##### Distribution.

*Pseudocerosrawlinsonae* has been recorded in the Caribbean Sea: Virgin Islands, Honduras, Jamaica, Bahamas, Curaçao; the Gulf of Mexico, Florida (Bolaños et al. 2007; Litvaitis et al. 2010, 2019); Brazil (Bahia et al. 2014, 2015; Bahia and Schrödl 2018). This is the first record for the coasts of Quintana Roo (Mexican Caribbean), Campeche and Yucatán, Mexico.

##### Description.

**Figure 9.**
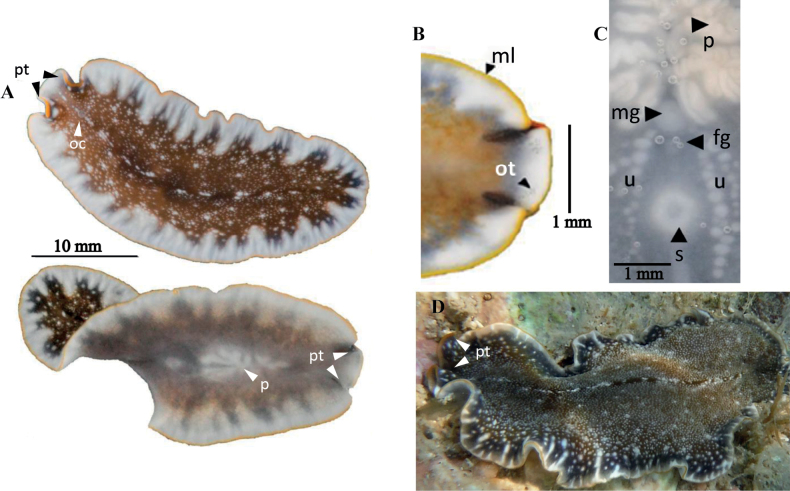
*Pseudocerosrawlinsonae***A** anterior end with pseudotentacles **B** dorsal y ventral view **C** ventral detail of pharynx, male and female gonopore, uteri and sucker **D***P.rawlinsonae* in situ.

Body shape elongated with rounded anterior and posterior end, 1.2 cm in length and 0.5 cm in width. Pigmentation brownish yellow to black with scattered white dots (Fig. 9A, D). A white marginal band with grey to black stripes encircles body margin. A characteristic golden yellow or orange marginal line marks external rim. Pseudotentacles simple folds (Fig. 9A, B). Cerebral eyes arranged in arrowhead shape, tentacular eyes more densely arranged along margins of pseudotentacles. Two frontal eye clusters positioned between two pseudotentacles. Ruffled pharynx in anterior region, oral pore situated centrally. Female and male gonopores separate and located in mid-region of the body, with sucker posterior to them (Fig. 9C).

##### Remarks.

*Pseudocerosbicolor* and *P.rawlinsonae* are closely related. Externally, the primary distinguishing features between the two are the prominent white submarginal band and the orange rim that characterise *P.rawlinsonae*. A study by Litvaitis et al. (2010) provided a detailed comparison between both species, examining both morphological and molecular data, specifically through analysis of the 28S gene.

#### ﻿ Pseudoceros bolool

Taxon classificationAnimalia﻿Polycladida﻿Pseudocerotidae

Newman & Cannon, 1994

E227B06F-459A-5DB2-91FF-641660633289

Fig. 10

##### Material examined.

**Campeche coast, Mexico** • 1; Cayo Arcas; 20.2°N, 92.0°W; 3.2 m; 24 Apr. 2018; A. Hernández leg.; CRPPY-0079.

##### Distribution.

Recorded in Heron Island and One Tree Island, Australia; Madang, Papua New Guinea (Newman and Cannon 1994); Shivrajpur, Gujarat (Thakkar et al. 2017); Andaman and Nicobar Island, India (Sreeraj and Raghunathan 2015); Caribbean Sea and Florida (Rawlinson 2008). This is the first record for the Campeche coast. New record for the Gulf of Mexico.

##### Description.

**Figure 10.**
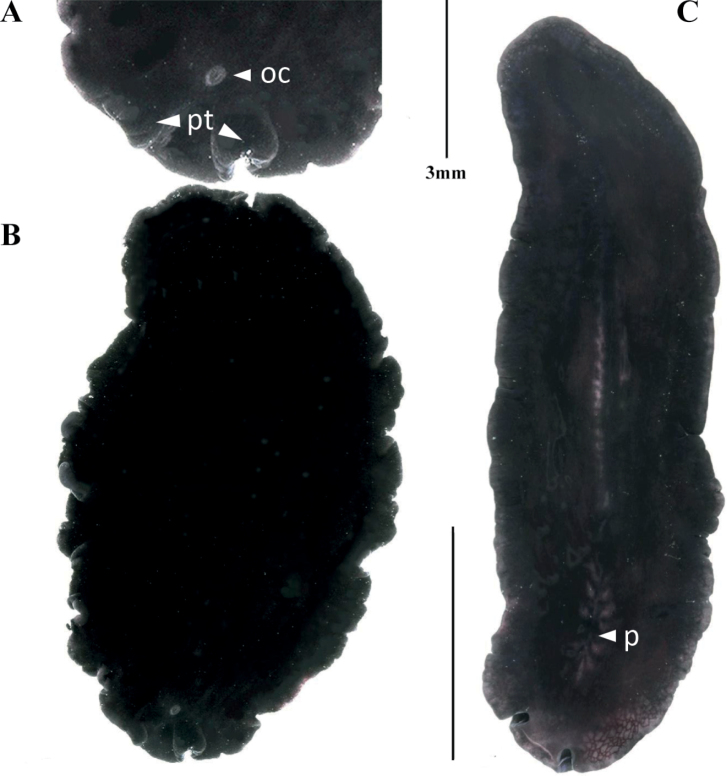
*Pseudocerosbolool***A** detail of the shape of the pseudotentacles and cerebral eyes **B** dorsal view **C** ventral view.

Body shape elongated with rounded anterior margin, tapering posteriorly, 3.5 cm in length and 1 cm in width. Margins slightly wavy. Ground colour velvety black, without any specific additional pattern, but with a small stain, devoid of pigment, present in area of cerebral eyes (Fig. 10A, B). Greyish ventrally. A characteristic bulge marks main intestinal trunk in body midline (Fig. 10C). Pseudotentacles simple folds of anterior margin. Cluster of cerebral eyes horseshoe-shaped at anterior end (Fig. 10A).

##### Remarks.

Within *Pseudoceros*, *P.bolool* and *P.velutinus* (Blanchard, 1847) share several external and internal morphological characters, characterised by a uniform velvety black coloration, without spots, bands, or marginal lines. While both species share several external and internal morphological traits, they can be distinguished by the ventral coloration: *P.velutinus* has a bluish violet background whereas *P.bolool* is grey, and by their marginal folds, which are broader in *P.velutinus* and more subtly defined in *P.bolool*. The specimen found in the Gulf of Mexico matches the original description of *P.bolool*. This species has been previously reported from the Australasian region (Newman and Cannon 1994, 1998) and the Indomalayan region (Dixit et al. 2021). Prior to this study, *P.bolool* was cited in Florida by Rawlinson (2008).

#### ﻿ Pseudoceros juani

Taxon classificationAnimalia﻿Polycladida﻿Pseudocerotidae

Bahia, Padula, Lavrado & Quiroga, 2014

B7346B3E-2705-5EAA-BA08-52669AD0601F

Fig. 11

##### Material examined.

**Campeche coast, Mexico** • 1; Cayo Arcas; 20.2°N, 92.0°W; 5 m; 26 Apr. 2018; A. Hernández leg.; CRPPY-0086.

##### Distribution.

Cabo Frío, Brazil (Bahia et al. 2014). First record for the Gulf of Mexico (Campeche, Mexico).

##### Description.

**Figure 11.**
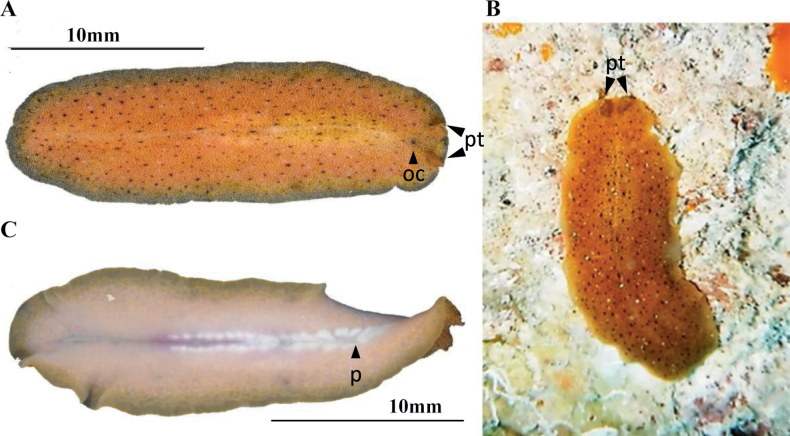
*Pseudocerosjuani***A** dorsal view **B** In situ **C** ventral view.

Elongated and elliptical body, 1.5 cm in length and 0.5 cm in width (Fig. 11A). Margin slightly wavy. Dorsal surface brick-orange with white dots and small black spots (Fig. 11A, B). Translucent whitish marginal band with a yellowish line visible (Fig. 11B). Pseudotentacles brick-orange, short, and as simple folds. Cluster of cerebral eyes horseshoe-shaped. Pseudotentacular eyes present. Two clusters of marginal eyes situated between pseudotentacles. Pharynx ruffled and butterfly-shaped (Fig. 11C).

##### Remarks.

Specimens of *Pseudocerosjuani* from Brazil show a darker colouration, characterised by more abundant and smaller dots distributed along the middle dorsal line. Additionally, the white marginal band with a yellow line is more conspicuous in the Brazilian exemplar. Disparities in the distribution of the dorsal dots and the lighter tones of the final brown band may be attributed to the maturity state of the individuals. Brazilian individuals are longer than those from the Gulf of Mexico.

### ﻿*Pseudobiceros* Faubel, 1983

#### ﻿ Pseudobiceros caribbensis

Taxon classificationAnimalia﻿Polycladida﻿Pseudocerotidae

Bolaños, Quiroga & Litvaitis, 2007

1AE5DEE3-0194-5438-A556-4A0A1AD95E30

Fig. 12

##### Material examined.

**Campeche coast, Mexico** • 1; Cayos sumergidos del Oeste; 20.4°N, 92.2°W; 13 m; 14 Sep. 2017; A. Hernández leg.; CRPPY-0014 • 1; Cayos sumergidos del Oeste; 20.4°N, 92.2°W; 10.8 m; 14 Sep. 2017; A. Gutiérrez leg.; CRPPY-0019 • 3; Cayo Arcas; 20.2°N, 92.0°W; 4.1 m; 21 Apr. 2018; A. Hernández leg.; CRPPY-0057 • 1; Cayo Arcas; 20.2°N, 92.0°W; 2.2 m; 21 Apr. 2018; A. Hernández leg.; CRPPY-0058 • 1; Cayo Arcas; 20.2°N, 92.0°W; 9.3 m; 24 Apr. 2018; A. Hernández leg.; CRPPY-0075 • 1; Cayo Arcas; 20.2°N, 92.0°W; 4.4 m; 17 Aug. 2018; A. Hernández leg.; CRPPY-0105.

##### Distribution.

Recorded in Curaçao, Jamaica, Florida, and Honduras (Bolaños et al. 2007); Belize (Rawlinson 2008). This is the first record for the Gulf of Mexico (Campeche coast, Mexico).

##### Description.

**Figure 12.**
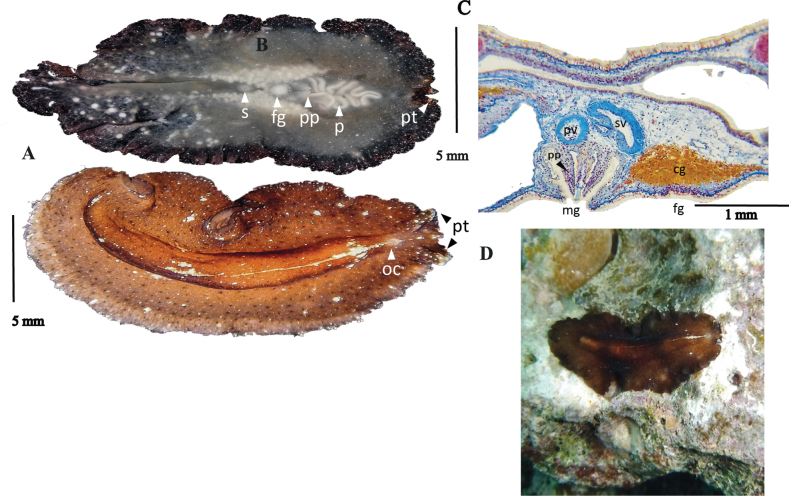
*Pseudobiceroscaribbensis***A** dorsal view **B** ventral view **C** sagittal section of the reproduction organs (stained with AZAN) **D** in situ.

Elongated and elliptical body, 2 cm in length and 1 cm in width, Dorsal background pigmentation caramel-brown with dispersed, darker tonalities. Small white and black spots scattered across entire surface. Median longitudinal thickening traversed with two large white patches, white median line more visible in anterior ½ of body, especially between patches. One patch situated near pharynx in first 1/3 of body, and second in posterior region of body (Fig. 12A, B, D). Ventral surface appears greyish with darker edges and dispersed white spots (Fig. 12B). Multiple marginal folds. Pseudotentacles complex with multiple folds. Cluster of cerebral eyes horseshoe shaped, situated in a pale rounded area. Ruffled pharynx located in first 1/3 of body, with a centred mouth. Two male copulatory openings in middle body region, behind the pharynx, located near female gonopore and sucker (Fig. 12C). Configuration of reproductive system matches that provided in the original description (Bolaños et al. 2007).

#### ﻿ Pseudobiceros splendidus

Taxon classificationAnimalia﻿Polycladida﻿Pseudocerotidae

(Lang, 1884)

2CDE5EF8-E8F4-516C-A1C2-88161D15F19A

Fig. 13

##### Material examined.

**Yucatan coast, Mexico** • 1; Bajos de Sisal; 21.2°N, 90.0°W; 1 m; 22 Feb. 2018; A. Hernández leg.; CRPPY-0030.

##### Distribution.

Originally described from Naples, Italy (Lang 1884). Recorded in Bermuda, Puerto Rico, Mid Turtle Shoal, Hawk Channel, Florida Keys, and the Atlantic coast of Florida, USA (Lang 1884; Hyman 1939, 1955; Litvaitis et al. 2019); Forte de Itaipú, Santos, São Paulo, Extremoz, Rio Grande de Norte, and Cabo Frío, Rio de Janeiro, Brazil (Marcus 1950; Bahia et al. 2012, 2014); Heron Island and One Tree Island, Great Barrier Reef, Australia; Hawaii, USA; Madang, Papua New Guinea; Rottnest Island, Western Australia; Andaman and Nicobar Islands, India; Indonesia; Maldives; South Africa; Singapore (Newman and Cannon 1994, 1997; Marquina et al. 2015; Litvaitis et al. 2019). New records for the Yucatan coast and Gulf of Mexico.

##### Description.

**Figure 13.**
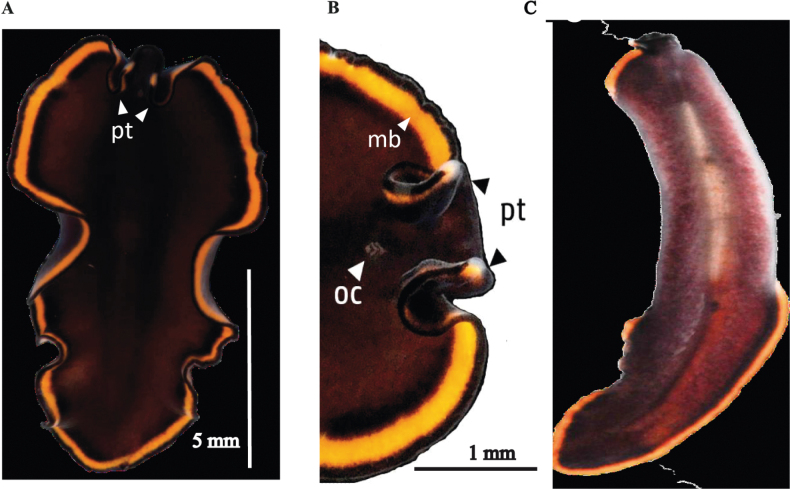
*Pseudobicerossplendidus***A** dorsal view **B** detail of colouration and marginal bands, pseudotentacles, and cluster of cerebral eyes **C** ventral view.

Body shape elongated with rounded anterior end and tapered posterior end, 1 cm in length and 0.5 cm in width. Velvety, wine-coloured background with a submarginal orange and marginal black band, interrupted at level of the pseudotentacles (Fig. 13A, C). Cerebral eyes located in a pigmentation-less area (Fig. 13B). Ruffled pharynx in the first 1/3 of the body, with the mouth. Two male copulatory organs are located close to the female gonopore and near the pharynx. Ventral sucker centred in the second corporal 1/3.

##### Remarks.

Specimens of *Pseudobicerossplendidus* studied show a colouration pattern similar to the specimens from Florida, illustrated in Litvaitis et al. (2019: fig. 9A). Litvaitis et al. (2019) grouped the closely related and similar species *Pseudobicerosevelinae*, *P.periculosus*, and *P.hymanae* into a single species, *P.splendidus*. This grouping is based on the results obtained through the molecular analyses of 28S and the few morphological differences found between these species (Litvaitis et al. 2019).

##### Biology.

The samples were collected under rocks associated with ascidians, possibly the primary food source of this species.

#### ﻿ Pseudobiceros pardalis

Taxon classificationAnimalia﻿Polycladida﻿Pseudocerotidae

(Verrill, 1900)

17A57FC0-DF88-55A0-B92E-61B2FCFE9A6F

Fig. 14

##### Material examined.

**Quintana Roo coast, Mexico** • 1; Mahahual; 18.6°N, 87.7°W; 5.3 m; 17 Mar. 2018; A. Hernández leg.; CRPPY-0113.

##### Distribution.

Original description from Bermuda (Verrill 1900); Bocas del Toro, Panamá, Caribbean Sea (Bolaños et al. 2007; Marcus 1950); Rio de Janeiro and Alagoas, Brazil (Bahia et al. 2012, 2014, 2015). New record for the cost of Quintana Roo (Mexican Caribbean).

##### Description.

**Figure 14.**
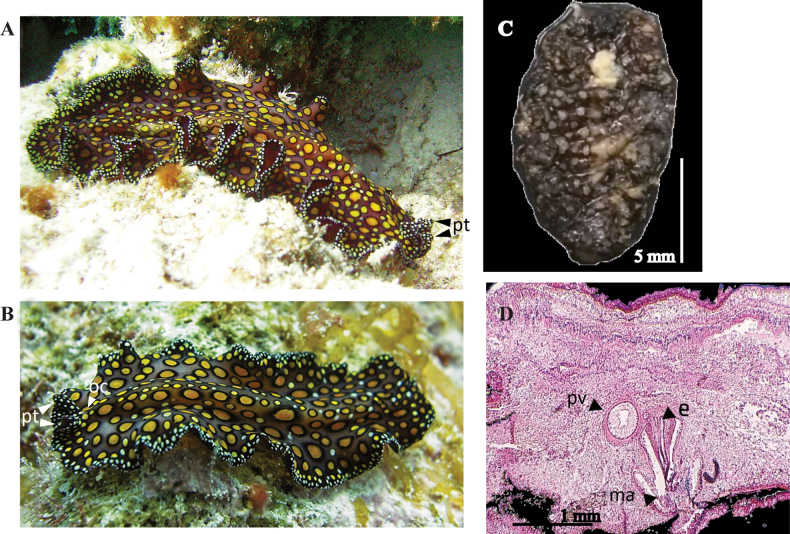
*Pseudobicerospardalis***A, B** photographes by Christine Loew and Matteo Cassela in Playa del Carmen (Mexico) **C** Specimen preserved for museum collections **D** sagittal section of the male reproductive system, prostatic vesicle stylet, male atrium (hematoxylin-eosin stain).

Body shape elongated with rounded anterior and tapering posteriorly, 1.3 cm in length and 0.7 cm in width. Body margins slightly wavy. Purple-brown background, darker at the margins, with yellow and orange spots outlined by a black circle, and tiny white spots along the entire body margin (Fig. 14A, B, C). Ventral surface characterised by a light purple shade, more translucent towards the margin. Two external and prominent male gonopores, together with the female gonopore located in the ventral midline (Fig. 14D). Additionally, a ventral sucker is present, situated in the centre of the body.

### ﻿*Phrikoceros* Newman & Cannon, 1996

#### ﻿ Phrikoceros mopsus

Taxon classificationAnimalia﻿Polycladida﻿Pseudocerotidae

(Marcus, 1952)

057B4FC6-B857-50EC-8BF3-18E523996BB4

Fig. 15

##### Material examined.

**Campeche coast, Mexico** • 1; Cayos sumergidos del Oeste; 20.4°N, 92.2°W; 13 m; 14 Sep. 2017; A. Gutiérrez leg.; CRPPY-0021; **Quintana Roo coast, Mexico** • 1; Mahahual; 18.6°N, 87.7°W; 13.6 m; 18 Mar. 2018; A. Hernández leg.; CRPPY-0043.

##### Distribution.

*Phrikocerosmopsus* was originally described in São Paulo, south-eastern Brazil (Marcus 1952). Later it was recorded in Antigua and Barbuda, Curaçao (Marcus and Marcus 1968); Argentina (Brusa et al. 2009; Bulnes et al. 2011); Brazil (Bahia et al. 2012, 2014, 2017; Bahia and Schrödl 2018); Colombia (Quiroga et al. 2004a); India (Sreeraj and Raghunathan 2015). This is the first record for the Campeche coast and Quintana Roo (Mexican Caribbean). New record for the Gulf of Mexico.

##### Description.

**Figure 15.**
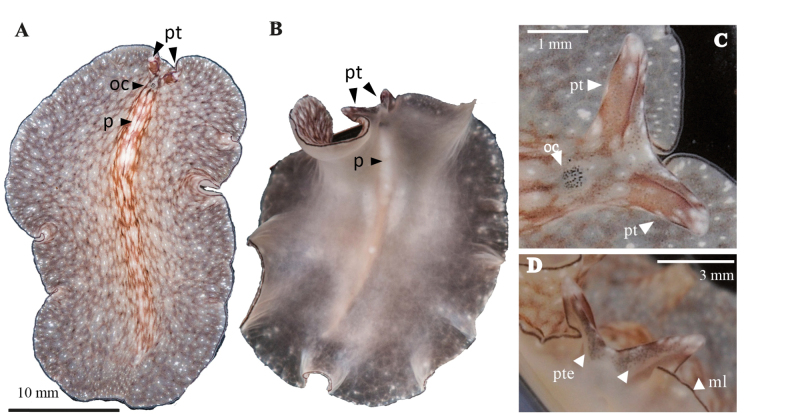
*Phrikocerosmopsus***A** dorsal view **B** ventral view **C** detail of the pseudotentacles and cerebral **D** marginal line and pseudotentacular eyes.

Body shape oval and elongated, with an extremely delicate consistency and a wavy margin, 3 cm in length and 1.5 cm in width. Marginal pseudotentacles deeply folded. Dorsally, with the characteristic small white spots on a caramel brown background, body midline darker. Marginal black rim, interrupted in the distal region of the pseudotentacles. (Fig. 15A, B). Two cerebral eyes clusters horseshoe-shaped and slightly separated. Pseudotentacular eyes grouped in two clusters placed ventrally and dorsally (Fig. 15C, D). Ventral surface beige (Fig. 15B). Ruffled pharynx and oral opening in the first 1/3 of the body, close to male and female gonopores. Ventral sucker in the middle of the body (Fig. 15B).

### ﻿*Thysanozoon* Grube, 1840

#### ﻿ Thysanozoon brocchii

Taxon classificationAnimalia﻿Polycladida﻿Pseudocerotidae

(Risso, 1818)

E9481E49-63F5-5375-82F5-A6E36D6B7859

Fig. 16

##### Material examined.

**Campeche coast, Mexico** • 1; Cayo Arcas; 20.2°N, 92.0°W; 6.3 m; 22 Apr. 2018; A. Hernández leg.; CRPPY-0068 • 1; Cayo Arcas; 20.2°N, 92.0°W; 13.2 m; 24 Apr. 2018; A. Hernández leg.; CRPPY-0076.

##### Distribution.

The species was described from Naples, Italy (Risso 1818). It is considered a cosmopolitan species, reported in the Mediterranean Sea, the United Kingdom, and southern and western Africa. In the western Atlantic, *Thysanozoonbrocchii* has been recorded in the Gulf of Mexico (Hyman 1952; Marcus and Marcus 1968); Caribbean coast of Colombia (Quiroga et al. 2004a, 2004b); Brazil (Bahia et al. 2014, 2015, 2017; Bahia and Schrödl 2018); Canary Islands (De Vera et al. 2009); Argentina (Brusa et al. 2009). In the Pacific it has been recorded in Japan and New Zealand (Prudhoe 1985). The record for Cayo Arcas, Campeche, represents the first record for Mexico.

##### Description.

**Figure 16.**
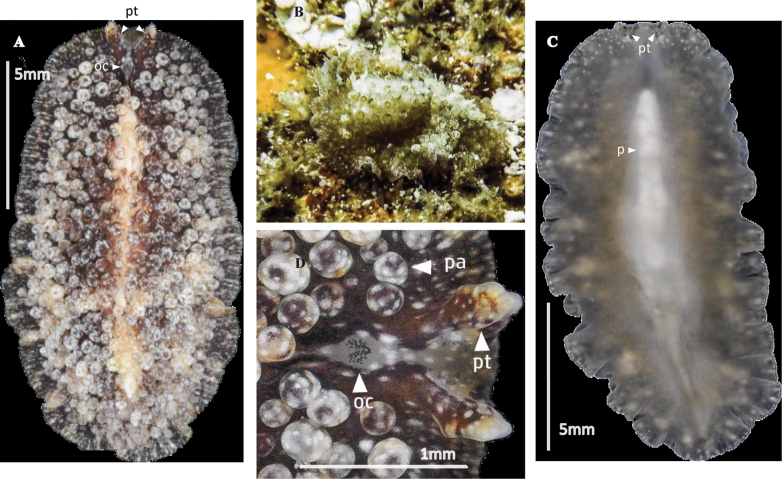
*Thysanozoonbrocchii***A** dorsal view **B** In situ **C** ventral view **D** detail of the dorsal surface; papillae, cerebral eyes, and pseudotentacles.

Body oval-shaped and firm consistency, 1.5 cm in length and 0.5 cm in width. Background colour ranges from brown to yellowish-ochre. Two stripes of paler cream spots, one longitudinally and the other perpendicular, form an inverted cross (Fig. 16A, B). The dorsal surface is covered with characteristic papillae, decreasing in size towards the margin. Pseudotentacles complex with multiple folds (Fig. 16D). Ruffled pharynx located in the first 1/3 of the body, with the oral opening in the middle. Two male copulatory apparatus and positioned close to the female organ in the ventral central region of the body. Sucker at the beginning of the second 1/2 of the body (Fig. 16C).

##### Remarks.

*Thysanozoonbrocchii* is noted for the abundance of papillae covering its dorsal surface and its cosmopolitan distribution. Various morphological descriptions with distinct colour patterns occur for different localities (Bahia et al. 2014). Molecular analysis of the different populations of this species is needed to identify potential divergences among the cited locations.

### ﻿Suborder Acotylea

**Discoceloidea Dittmann, Cuadrado, Aguado, Noreña, & Egger, 2019**

**Cryptocelididae Laidlaw, 1903**

***Phaenocelis* Stummer-Traunfels, 1933**

#### ﻿ Phaenocelis medvedica

Taxon classificationAnimalia﻿PolycladidaCryptocelididae

Marcus, 1952

0771719D-9953-56EB-9EEB-3D052ABDE0A9

Fig. 17

##### Material examined.

**Campeche coast, Mexico** • 1; Cayo Arcas; 20.2°N, 92.0°W; 6.2 m; 20 Aug. 2018; A. Hernández leg.; CRPPY-0109 • 1; Cayo Arcas; 20.2°N, 92.0°W; 3.3 m; 20 Aug. 2018; A. Hernández leg.; CRPPY-0112.

##### Distribution.

*Phaenocelismedvedica* was recorded in Brazil (Marcus 1952; Bahia et al. 2015; Bahia and Schrödl 2018); Caribbean coast of Colombia (Quiroga et al. 2004a, 2004b). New record for the Campeche coast and Gulf of Mexico.

##### Description.

**Figure 17.**
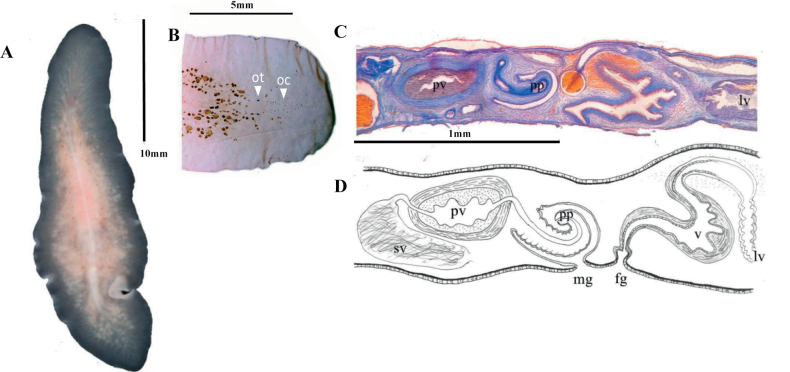
*Phaenocelismedvedica***A** dorsal view **B** detail of the marginal, cerebral and tentacular ocelli **C** sagittal section of the reproductive system **D** sagittal reconstruction of male and female apparatus.

Body shape elongated with rounded anterior end and pointed posterior end, 0.45 cm in length and 0.2 cm in width. Translucent pinkish colouration, including two longitudinal dark brown rows parallel to the main body’s axis (Fig. 17A). Two elongated clusters of cerebral eyes and two small groups of tentacular eyes sparsely distributed. Small marginal eyes along the entire body margin. (Fig. 17B). Pharynx central, occupies 1/3 of the body size. Male and female reproductive organs located in the second 1/2 of the body with the morphological features of *P.medvedica*. ***Reproductive system*.** The male reproductive system consists of a large and muscular interpolated prostatic vesicle, a slightly muscular seminal vesicle, and a large, coiled cirrus. Female copulatory organ with a bulbous vagina and large Lang’s vesicle (Fig. 17C, D).

#### ﻿ Phaenocelis peleca

Taxon classificationAnimalia﻿PolycladidaCryptocelididae

Marcus & Marcus, 1968

243274CC-5C58-522E-A539-76136A97F033

Fig. 18

##### Material examined.

**Campeche coast, Mexico** • 2; Cayo Arcas; 20.2°N, 92.0°W; 2.2 m; 20 Apr. 2018; A. Hernández leg.; CRPPY-0053 • 1; Cayo Arcas; 20.2°N, 92.0°W; 9.3 m; 24 Apr. 2018; A. Hernández leg.; CRPPY-0077.

##### Distribution.

Piscadera Bay, Curaçao, Caribbean Sea (Marcus and Marcus 1968). New record from the Campeche coast and Gulf of Mexico.

##### Description.

**Figure 18.**
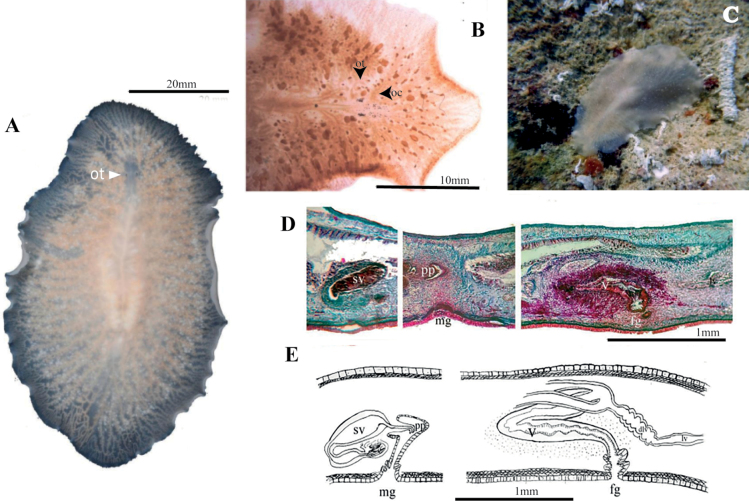
*Phaenocelispeleca***A** live animal photographed on black background **B** anterior end with tentacular eyes and cerebral eyes **C** in situ **D** sagittal section of the reproductive system **E** sagittal reconstruction of male and female copulatory organs.

Body shape oval and tapers in the posterior region, 4 cm in length, 2 cm in width. Compact consistency. Milky-translucent colouration, with translucent margin (Fig. 18A, C). Intestinal branches visible through transparency. Well-differentiated tentacular eyes; cerebral eyes in scattered, elongated clusters; abundant marginal eyes along the entire body margin (Fig. 18B). Pharynx ruffled, elongated and narrow. Male and female gonopores close to the oral pore. ***Reproductive system*.** Male reproductive system (Fig. 18D, E) consists of a short, curved penis papilla, an elongated prostatic vesicle, and a short seminal vesicle with distally broad seminal ducts. Female reproductive system, poorly developed in the studied specimens (Fig. 18D, E), consists of an elongated vagina externa, surrounded by cement cells, a vagina interna and Lang’s vesicle.

##### Remarks.

The specimen of the Gulf of Mexico aligns with the description of *Phaenocelispeleca* provided by Marcus and Marcus (1968). Nonetheless, Mexican *P.peleca* specimens are larger than Caribbean ones (4 cm vs 2 cm), but present a short penis papilla compared to that given in the original description. Female apparatus differences are also observed, with the Cayo Arcas *P.peleca* having an elongated Lang’s vesicle compared to the round one in Caribbean individuals. These differences may be due to specimen size and maturity.

### ﻿Leptoplanoidea Faubel, 1984

**Stylochoplanidae Faubel, 1983**

***Stylochoplana* Stimpson, 1857**

#### ﻿ Stylochoplana sisalensis sp. nov.

Taxon classificationAnimalia﻿PolycladidaStylochoplanidae

C410244E-197C-5916-A288-C43254621CC2

https://zoobank.org/962C25B2-2F1B-4A9F-8897-B458D326398A

Fig. 19

##### Material examined.

***Holotype*: Campeche coast, Mexico** • 1; 9 slides; Cayos sumergidos del Oeste; 20.4°N, 92.2°W; 0 m; 11 Sep. 2017; A. Hernández leg.; CRPPY-0013. ***Paratypes*: Campeche coast, Mexico** • 1; Cayo Arcas; 20.2°N, 92.0°W; 6.2 m; 20 Aug. 2018; A. Hernández leg.; CRPPY-0110 • 1; Cayo Arcas; 20.2°N, 92.0°W; 3.3 m; 20 Aug. 2018; A. Hernández leg.; CRPPY-0111.

##### Distribution.

Found in submerged West Keys of Reef Triángulos and Cayo Arcas, Campeche coast, Mexico.

##### Description.

**Figure 19.**
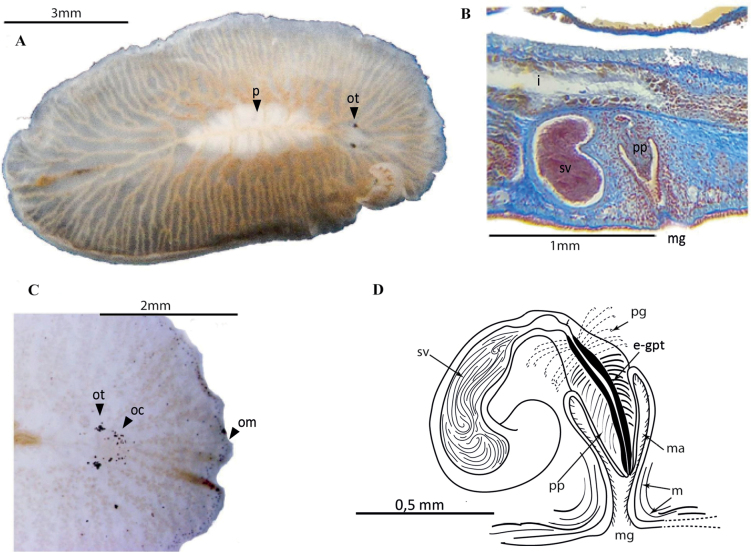
*Stylochoplanasisalensis* sp. nov. **A** dorsal view **B** sagittal section of a male copulatory organ without a prostatic vesicle, only showing a penis papilla **C** anterior end with cerebral and marginal eyes **D** sagittal reconstruction of the reproductive system.

Body shape oval with rounded anterior and posterior end, 10 mm long and 5 mm wide. Whitish translucent colour with a pale brown tonality due to the gut contents. A network of independent intestinal branches, not anastomosing, extends to the body’s margin (Fig. 19A). Presence of two compact clusters tentacular eyes (12–17 eyes per cluster), two scattered clusters of cerebral eyes (15–20 eyes per cluster, distributed within 0.03 mm in front of the tentacular eyes) and some marginal eyes in the frontal region (Fig. 19B). Ruffled pharynx located in the second 1/3 of the body. ***Reproductive system*.** Testes dorsal and ovaries ventral. Seminal vesicle well developed, elongated and wide surrounded by thin muscular walls. The ejaculatory duct runs upward, backward, and then curves downward before widening as it enters the penis papilla. Lining of walls of this internal dilation forms epithelial glandular prostate tissue which functions as a prostatic vesicle. The glandular prostate epithelium stores the secretion from the extra-vesicular glands (Fig. 19B, D). Conical, naked penis papilla (without a stylet) covered by a non-ciliated, flat epithelium. It projects into a deep male atrium, with a tall ciliated epithelium (Fig. 19C). The female copulatory organ was barely developed in the only specimen observed, so it could not be described in detail.

##### Etymology.

The name *sisalensis* is dedicated to the town where the research centre is located, the UNAM campus in Sisal, Yucatán province, Mexico.

##### Remarks.

Currently, the genus *Stylochoplana* comprises 25 valid species worldwide. This genus is one of the most species-rich within the order Polycladida and has been divided into different informal groups by several authors (Bock 1913; Marcus and Marcus 1968) since it was described by Stimpson (1857). *Stylochoplanasisalensis* is included in group B of Bock (1913) or B1 of Marcus and Marcus (1968), characterised by tentacles absent, unarmed papilla peneal, absence of penial pocket, and Lang’s vesicle present. This group includes the following species:

- *S.chilensis* (Schmarda, 1859): with epithelial-glandular prostate tissue (Stummer-Traunfels 1933).
- *S.chloranota* (Boone, 1929): with interpolated prostatic vesicle (Hyman 1953).
- *S.graffi* (Laidlaw, 1906): with interpolated prostatic vesicle (Bock 1913).
- *S.longipenis* Hyman, 1953: with interpolated prostatic vesicle.
- *S.minuta* Hyman, 1959: with epithelial-glandular prostate tissue, but forms a receptacle or container in the proximal region of the papilla peneal.
- *S.nadiae* (Melouk, 1941): without data.
- *S.suosensis* Kato, 1943: with epithelial-glandular prostate tissue. The female apparatus is not known, and so it is unknown whether the species belongs to B1 (with Lang’s vesicle) or B2 (without Lang’s vesicle).
- *S.utunomii* Kato, 1943: with epithelial-glandular prostate tissue.
- *S.walsergia* Marcus & Marcus, 1968 (no. 12): with epithelial-glandular prostate tissue.

*Stylochoplanasisalensis* sp. nov. presents the greatest similarity with *S.walsergia* from Brazil, *S.chilensis* from Chile, *S.utunomii* from Japan, and *S.minuta* from the Palau Islands (Micronesia). These species are all characterised by the presence of a well-developed and elongated seminal vesicle, as well as an ejaculatory duct that widens and is covered by a prostatic glandular epithelium. All other species in this group present an isolated, more or less elongated and interpolated prostatic vesicle.

On the other hand, the species of Marcus’ Group BI present a very similar female copulatory apparatus directed towards the anterior region and, at the level of the internal vagina, then curving towards the posterior region. In the middle of the female duct, the oviduct opens and the internal epithelium thickens to form Lang’s duct that ends in the rounded Lang’s vesicle.

*Stylochoplanasisalensis* differs from *S.walsergia* by the location of the prostate tissue and the shape of the penis papilla. In *S.walsergia*, the prostatic dilation is included entirely in the penis papilla and surrounded by the male atrium, while in *S.sisalensis* the penis papilla encloses only 1/2 of the prostatic tissue and the common male duct. This characteristic is shared by *S.chilensis*, but not with *S.utunomii* in which the prostate tissue is practically outside the penis papilla, a short protrusion within the male atrium. As in *S.suosensis*, we lack data on the female apparatus, but we assume that its arrangement is like that of the entire Marcus group B of *Stylochoplana*.

### ﻿*Emprosthopharynx* Bock, 1913

#### ﻿ Emprosthopharynx hartei sp. nov.

Taxon classificationAnimalia﻿PolycladidaStylochoplanidae

96B096CE-2EB1-583D-A836-B9D4C744CEE8

https://zoobank.org/0E7C472E-3BC9-4326-916A-CF91C641F5D1

Fig. 20

##### Material examined.

***Holotype*: Yucatan coast, Mexico** • 1; 18 Slides; Dzilam; 21.5°N, 88.9°W; 13 m; 10 May 2018; A. Hernández leg.; CRPPY-0095. ***Paratypes*: Yucatan coast, Mexico** • 4; Dzilam; 21.5°N, 88.9°W; 14 m; 9 May 2018; A. Hernández leg.; CRPPY-0092 • 3; Dzilam; 21.5°N, 88.9°W; 14 m; 9 May 2018; A. Hernández leg.; CRPPY-0093.

##### Distribution.

Dzilam de Bravo, Yucatan, Mexico.

##### Description.

**Figure 20.**
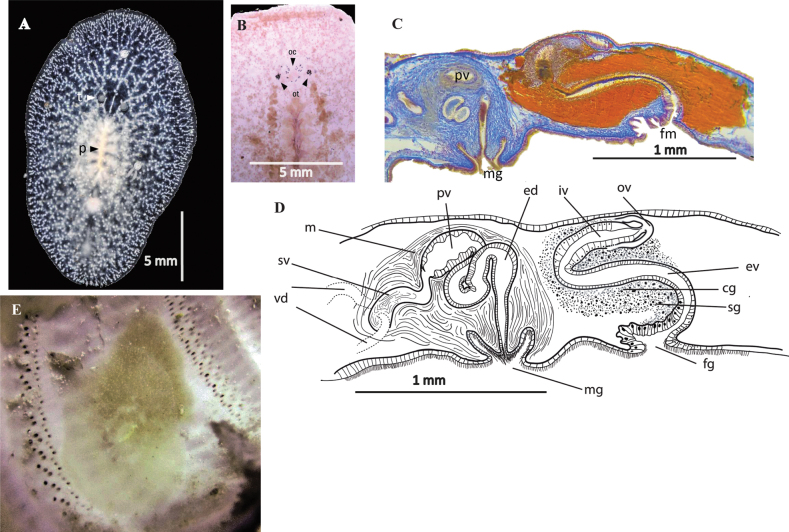
*Emprosthopharynxhartei* sp. nov. **A** photographed on a black background, where the intestinal branches and pharynx are patent **B** anterior end with tentacular eyes and cerebral eyes **C** histological sagittal section (Azan stained) at the level of the reproductive system **D** sagittal reconstruction of the reproductive system **E** specimen in situ, showing natural colouration. Abbreviations: ot tentacular eyes, oc cerebral eyes

Body shape elliptical, 10 mm length and 7 mm width. Body constitution solid with a translucent background (Fig. 20A). Conspicuous whitish intestinal branches extend towards margins. Body periphery with a white dotted line. Nuchal tentacles rounded and prominent, with 20–30 eyes per tentacle. Two small clusters of cerebral eyes close to the tentacles are, 12–20 eyes per cluster (Fig. 20B). Ruffled pharynx centrally positioned. Oral pore located at pharynx centre. Dorsal pore dorsally in the last body 1/3, visible when examined in vivo (Fig. 20A, E). ***Reproductive system*.** Male and female copulatory apparatus situated posterior to the pharynx, in the second 1/2 of the body. Male copulatory organ immersed in a muscular bulb: the seminal vesicle, prostatic vesicle, ejaculatory duct, and an elongated penis papilla covered with long cilia with a sclerotised appearance. Elongated seminal vesicle receives the vasa differentia separately at its proximal end. Distally the seminal vesicle connects to the prostatic vesicle through a narrow duct. Prostatic vesicle lined with a thick wavy epithelium and extending into a long duct that surrounds the ejaculatory duct. The ejaculatory duct is ciliated and discharges in the penis papilla. A narrow and shallow male atrium houses the penis papilla (Fig. 20C, D). Female reproductive system characterised by a short, wide, muscular atrium covered by a well-developed ciliated epithelium. A narrow tubular vagina externa leads from the atrium towards the wider vagina interna, lined with glandular epithelium. At its distal end, the vagina interna divides into two oviducts that turn towards the anterior region. Both sections of the vagina, but especially the vagina interna, are surrounded by dense masses of cement and shell glands. Lang’s vesicle is absent (Fig. 20C, D).

##### Etymology.

The species name *hartei* is dedicated to the conservationist Edward H. Harte, in recognition of his lifelong commitment to environmental conservation and his significant contributions to marine science and the protection of marine ecosystems.

##### Remarks.

Currently, the genus *Emprosthopharynx* is composed of nine species: *E.gracilis* (Heath & McGregor, 1912); *E.hancocki* (Hyman, 1953); *E.heroniensis* Beveridge, 2018; *E.lysiosquillae* Oya, Nakajima & Kajihara, 2022; *E.opisthoporus* Bock, 1913; *E.pallida* (Quatrefage, 1845); *E.vanhoffeni* Bock, 1931; *E.onubensis* Pérez-García, Gouveia, Calado, Noreña & Cervera, 2024; and *E.rasae* Prudhoe, 1968. The genus is distributed mainly within the Pacific Ocean, except for *E.pallida* and *E.onubensis*, which are native to the Mediterranean, and *E.vanhoffeni* found in the Cape Verde Islands (Bock 1931) and Morocco (Prudhoe 1985). *Emprosthopharynxhartei* sp. nov. lacks marginal eyes as do *E.pallida*, *E.onubensis*, *E.hancocki*, *E.gracilis*, and *E.heroniensis*. Still, it can be distinguished from the other *Emprosthopharynx* species by the presence of tentacles, which *E.hartei* shares only with *E.hancocki* and *E.gracilis* (Pérez-García et al. 2024).

Regarding the internal characteristics between *Emprosthopharynxhartei* and *E.hancocki*, in both species, the distal region of the papilla peneal is covered by a series of bristles, thickened, or with pseudosclerotised cilia (the styliform development of the basal membrane mentioned by Faubel 1983, 1984). This pseudosclerotised formation differentiates these two species from the other species of *Emprosthopharynx*, which either present a true stylet (*E.vanhoffeni* and *E.lysiosquillae*) or show a naked papilla peneal, without hard structures (*E.heronensis*, *E.gracilis*, *E.onubensis*, *E.opisthoporus*, *E.pallidus*, and *E.rasae*).

The main difference between *Emprosthopharynxhancocki* and *E.hartei* is found at the level of the arrangement and shape of the reproductive system. The seminal vesicle and the prostatic vesicle in *E.hancocki* barely present a small constriction between one organ and another, while in *E.hartei* the transition between the seminal vesicle and the prostatic vesicle is marked by a tube-like narrowing. In addition, the prostatic vesicle in *E.hartei* empties through an elongated sinuous extension until the papilla peneal.

The distinction between *Emprosthopharynxgracilis* and *E.hartei* lies in the structural and morphological characteristics of the distal region of the male copulatory apparatus. *Emprosthopharynxhartei* has an elongated prostatic vesicle that leads to a long ejaculatory duct. The duct ends in a papilla peneal surrounded by a flattened atrium. In contrast, *E.gracilis* is characterised by a rounded prostatic vesicle and a short and robust papilla peneal that opens into a long and deep atrium. Within the female reproductive system, we can observe differences between *E.hartei* and *E.gracilis*. For instance, the female atrium in *E.hartei* is elongated and narrow, whereas in *E.gracilis* it is short and widened. Additionally, the thickening of the vagina is distinct in the two species: in *E.hartei*, the thickening is located in the proximal region, while in *E.gracilis* it is found in the distal region.

### ﻿Notoplanidae Marcus & Marcus, 1966

***Notoplana* Laidlaw, 1903**

#### ﻿ Notoplana annula

Taxon classificationAnimalia﻿Polycladida﻿Notoplanidae

Marcus & Marcus, 1968

EE31645C-6BF5-5E78-B2FC-FB03837D963B

Fig. 21

##### Material examined.

**Campeche coast, Mexico** • 5; Cayos sumergidos del Oeste; 20.4°N, 92.2°W; 5 m; 10 Sep. 2017; A. Hernández leg.; CRPPY-0009 • 8; Cayos sumergidos del Oeste; 20.4°N, 92.2°W; 0 m; 10 Sep. 2017; A. Gutiérrez leg.; CRPPY-0010 • 1; Cayos sumergidos del Oeste; 20.4°N, 92.2°W; 0 m; 11 Sep. 2017; A. Hernández leg.; CRPPY-0012 • 5; Cayos sumergidos del Oeste; 20.4°N, 92.2°W; 11 m; 11 Sep. 2017; A. Gutiérrez leg.; CRPPY-0016 • 2; Cayos sumergidos del Oeste; 20.4°N, 92.2°W; 13 m; 14 Sep. 2017; A. Gutiérrez leg.; CRPPY-0017 • 1; Cayos sumergidos del Oeste; 20.4°N, 92.2°W; 13 m; 14 Sep. 2017; A. Gutiérrez leg.; CRPPY-0023.

##### Distribution.

Recorded in Piscadera Bay and Fuik Bay, Curaçao and Virginia Key, Florida (Marcus and Marcus 1968). New record in the Gulf of Mexico: Campeche, Mexico.

##### Description.

**Figure 21.**
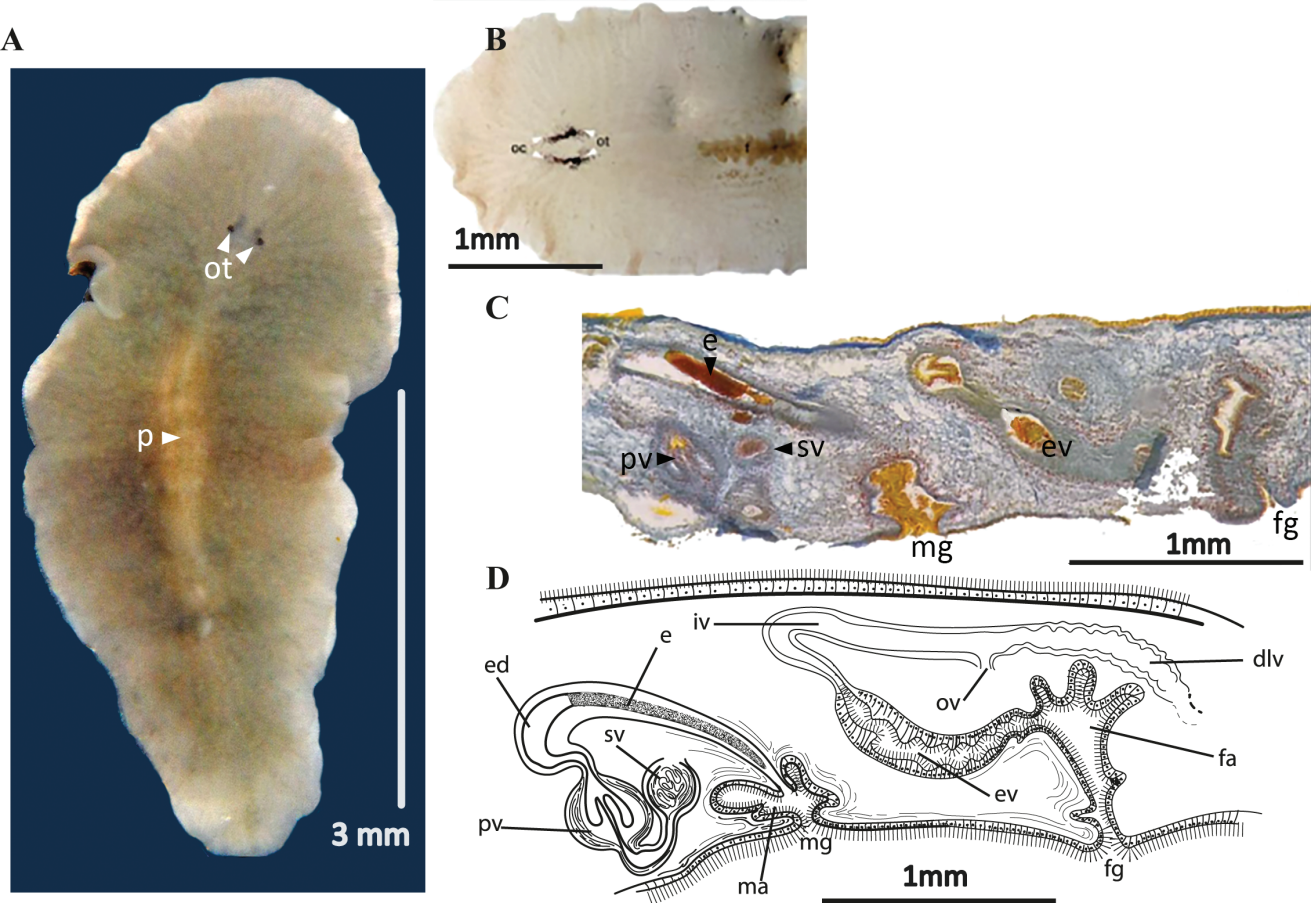
*Notoplanaannula***A** dorsal view **B** detail of cerebral and tentacular eyes **C** histological section of male and female copulatory organs **D** sagittal reconstruction of the reproductive system.

Body shape elongated and smooth, 0.7 cm in length and 0.3 cm in width. Pigmentation varies from whitish beige to greenish hues. Intestinal extensions well-branched, not anastomosing, extending to body margin, with contents visible due to transparency (Fig. 21A). Tentacular eyes form rounded cluster located in the brain, crossed by two, parallel, elongated clusters of cerebral eyes. Marginal eyes absent (Fig. 21B). Pharynx elongated, tapering along midsection of body. Male and female gonopores positioned posterior to the pharynx in the second 1/2 of the body.

Heading as above. Male copulatory system comprises an elongated seminal vesicle with two broad sperm ducts proximally (Fig. 21C, D). Prostatic vesicle interpolated, connected via a narrowing to the ejaculatory duct and the penis papilla. Ejaculatory duct with thin, long, curved stylet covered with a penial sheath. Atrium wide proximally and tubular distally. Female apparatus comprises a wide, densely ciliated female antrum, a ciliated external vagina with a sinuous course, the internal vagina, and Lang’s vesicle. Cement glands open into the vagina externa and oviducts into the vagina interna (Fig. 21C, D).

### ﻿Notocomplanidae Litvaitis, Bolaños & Quiroga, 2019

***Notocomplana* Faubel, 1983**

#### ﻿ Notocomplana ferruginea

Taxon classificationAnimalia﻿Polycladida﻿Notocomplanidae

(Schmarda, 1859)

F4D23C40-9212-5D89-AD3A-A8E8C6FBCA40

Fig. 22

##### Material examined.

**Yucatan coast, Mexico** • 1; Arrecife Alacranes; 22.4°N, 89.7°W; 1 m; 4 Nov. 2017; A. Hernández leg.; CRPPY-0004; **Veracruz coast, Mexico** • 6; Veracruz; 19.2°N, 96.1°W; 1 m; 31 Sep. 2018; A. Hernández leg.; CRPPY-0101 • 7; Veracruz; 19.2°N, 96.1°W; 2 m; 1 Sep. 2018; A. Hernández leg.; CRPPY-0103.

##### Distribution.

The species was described from Jamaica (Schmarda 1859); Colombia, Antilles, and Bahamas (Hyman 1955; Marcus and Marcus 1968; Quiroga et al. 2004a, 2004b). New record for the coasts of Veracruz and Yucatán. New record for the Gulf of Mexico.

##### Description.

**Figure 22.**
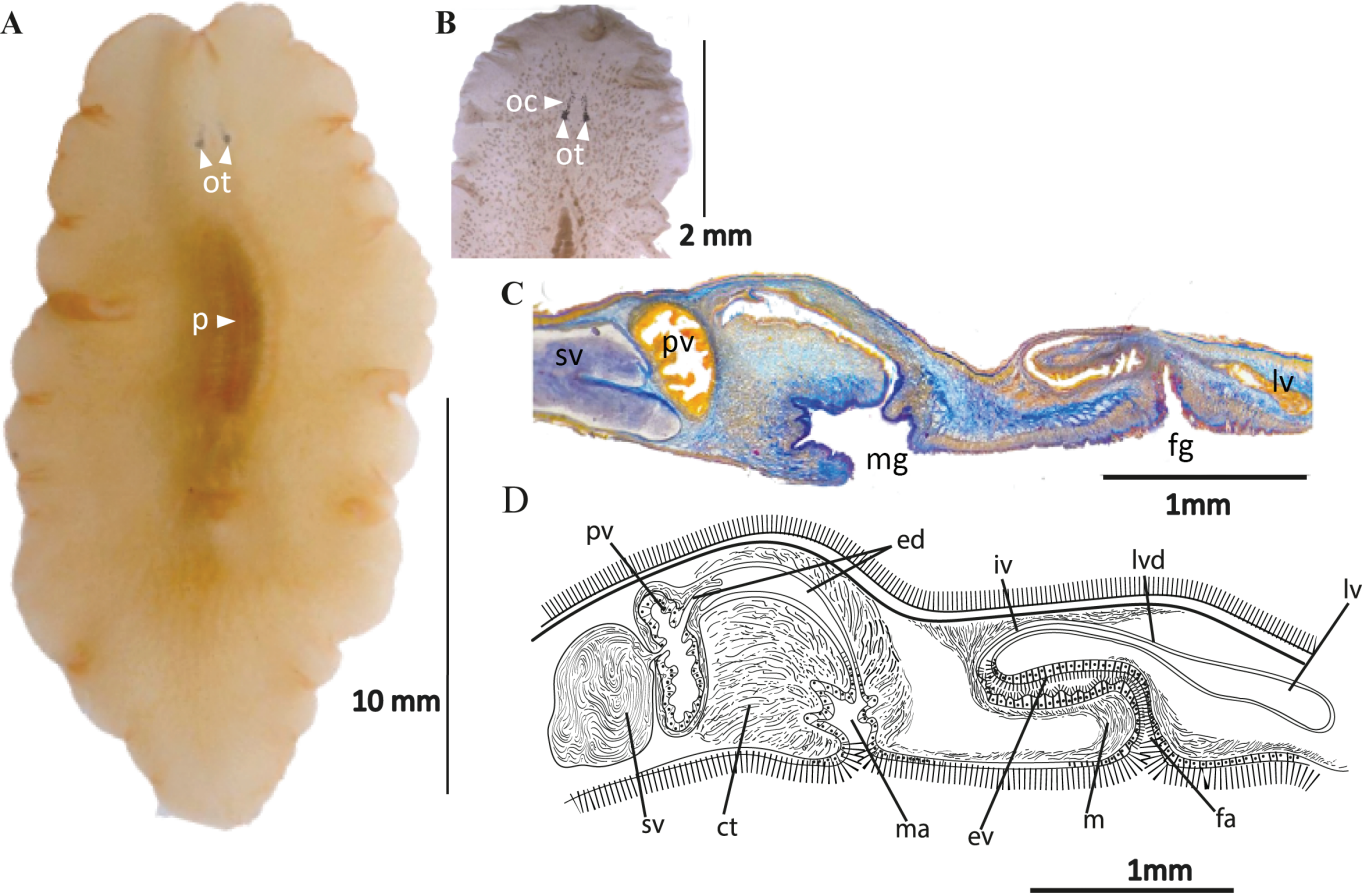
*Notocomplanaferruginea***A** dorsal view **B** approach to the tentacular, cerebral and ruffled pharynx **C** sagittal section of the reproductive system **D** sagittal reconstruction of male and female apparatus.

**Figure 23.**
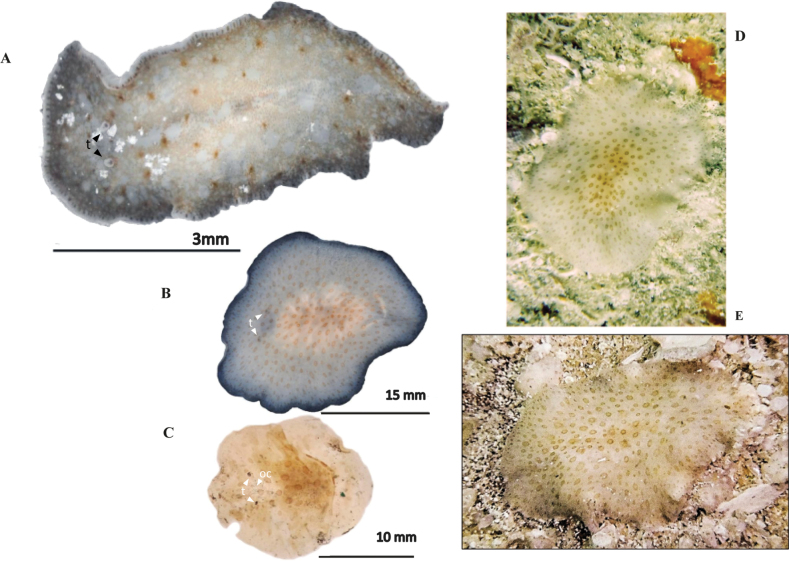
**A***Gnesiocerossargassicola* Photograph in situ of the specimen captured **B–E***Idioplanaatlantica***B** dorsal view of a live individual photographed on a black background **C** specimen photographed after fixation **D, E** specimens photographed in situ with the characteristic pigmentation rings.

Body shape oval with rounded anterior and tapered posterior end, margins pale, wavy, 2.5 cm in length and 1 cm in width. Translucent beige colouration, darker in the middle region of the pharynx (Fig. 22A). Two groups of well-defined cerebral and tentacular eyes (Fig. 23B). No marginal eyes. A short stylet is present. Prostatic vesicle interpolated. Male atrium very muscular. An elongated atrium, a muscular vagina externa and interna as well as Lang’s vesicle form the female copulatory apparatus (Fig. 22C, D).

##### Remarks.

Research conducted by Oya and Kajihara (2017) and Litvaitis et al. (2019) has led to the transfer of species within *Melloplana*, a genus belonging to the family Pleioplanidae, to the genus *Notocomplana* (family Notoplanidae). This transfer is based on analysis of the mitochondrial gene Cox1 (Oya and Kajihara 2017) and the nuclear gene 28S (Litvaitis et al. 2019). Only a few morphological differences between *Melloplana* and *Notocomplana* have been identified. The main distinction lies in the orientation of the prostatic vesicle chambers: *Melloplana* prostatic chambers are perpendicular to the intra-vesicular ejaculatory duct, whereas *Notocomplana* chambers are longitudinally arranged (Faubel 1983). However, both *Melloplana* and *Notocomplana* lack a stylet.

Considering the limited morphological variation between the genera and recent molecular analyses, there is a tendency to propose eliminating the genus *Melloplana*, as well as the family Pleioplanidae. However, Pleioplanidae also includes other genera like *Izmira*, *Pleioplana*, and *Laqueusplana*, which are poorly known. Therefore, additional molecular analyses and morphological evidence are necessary to confirm the elimination of the family Pleioplanidae (Dittmann et al. 2019).

### ﻿Gnesiocerotidae Marcus & Marcus, 1966

***Gnesioceros* Diesing, 1862**

#### ﻿ Gnesioceros sargassicola

Taxon classificationAnimalia﻿Polycladida﻿Gnesiocerotidae

Mertens, 1832

F1CC6F2F-6D66-5DF9-BBCF-CD58A1A0F136

Fig. 23A

##### Material examined.

**Quintana Roo coast, Mexico** • 2; Mahahual; 18.6°N, 87.7°W; 7.7 m; 17 Mar. 2018; A. Hernández leg.; CRPPY-0037.

##### Distribution.

Currently, *Gnesiocerossargassicola* is known from Bermuda (Hyman 1939) and Florida to Texas, Gulf of Mexico US coast (Hyman 1954); on *Sargassum* algae, near the west coast of Africa (Moseley 1877); Boa Vista Island, Cape Verde (Laidlaw 1903); Gaira Bay, Colombia (Quiroga 2008); Curaçao, Bonaire, Little Bonaire, (Netherlands Antilles, Caribbean), Saint Barthelemy, France Antilles; Bahia Fosforescente, Puerto Rico; Marine Biological Station, Virginia Key, Florida, USA; Central Atlantic Ocean, Sargasso Sea (Marcus and Marcus 1968). New record for the coasts of Mahahual, Quintana Roo (Mexican Caribbean).

##### Description.

Dorsoventrally flattened body, anteriorly widened with a shallow constriction after the tentacles, undulated body margins, and a blunt-tailed posterior end, 0.8 cm in length and 0.3 cm in width. Yellowish grey background colouration with numerous, rounded, orange or brown spots. Narrow elongated pharyngeal pouch with a central oral opening (Fig. 23A).

##### Biology.

Associated with *Sargassum* algae.

### ﻿Stylochoidea Poche, 1926

**Idioplanidae Dittmann, Cuadrado, Aguado, Noreña & Egger, 2019**

***Idioplana* Woodworth, 1898**

#### ﻿ Idioplana atlantica

Taxon classificationAnimalia﻿PolycladidaIdioplanidae

(Bock, 1913)

B13A31A1-86A7-586F-AB65-6C06E450124A

Fig. 23B–E

##### Material examined.

**Yucatan coast, Mexico** • 3; Dzilam; 21.5°N, 88.9°W; 13 m; 10 May 2018; A. Hernández leg.; CRPPY-0096.

##### Distribution.

*Idioplanaatlantica* was originally recorded off St Thomas Island (USA Virgin Island, Caribbean Sea; Bock 1913). Similar morphotypes of this species have been reported in Bocas del Toro (Panama, Caribbean Sea; Litvaitis 2014–2024; Quiroga et al. 2004b); Aguadores Beach near Santiago de Cuba (Caribbean Sea; Catalá et al. 2016). New record for the coasts of Yucatán, Mexico.

##### Description.

Body shape oval, with a rounded posterior end and a more pointed anterior end, 2 cm in length and 1 cm in width. Firm and dense consistency. Background colour ranges from yellowish-white to amber. Dorsally, is covered by dark rings with cream-pigmented inner. The shape of these rings is variable, appearing more rounded anteriorly and elongated posteriorly. Also, the central rings are larger compared to those along the body’s margin (Fig. 23B, C, D, E). Near the anterior end, two cylindrical nuchal tentacles are present. Tentacular eyes immersed along the tentacles (Fig. 23E). Two elongated scattered clusters of cerebral eyes and the marginal eyes limited to the anterior of the body.

##### Remarks.

Yucatan specimens show a resemblance to those documented by Litvaitis (2014–2024), as well as by Kate Rawlinson in Bocas del Toro, Panama (https://www.invertebase.org/portal/taxa/index.php?taxauthid1&taxon=146957&clid=57). In these instances, organisms identified as *Idioplanaatlantica* (Bock, 1913) exhibit rounded dark rings. However, the type description of *Idioplanaatlantica*, based on fixed material, does not mention rounded spots or rings. The specimen documented by Bock (1913) exhibits a homogeneous yellowish colouration with a slightly orange tone in the central region, while the ventral side is white-grey. Also, the marginal eyes encircle the entire body edge, and the cerebral eyes are dispersed but abundantly present in the brain region. The living specimen and preserved Yucatan specimen displays dark rings covering its dorsal surface. Furthermore, marginal eyes are either absent or barely noticeable on the anterior end. The absence of pigmentation noted by Bock could be attributed to the fixation process. This differs from the Mexican material, where pigmentation remains intact throughout fixation processing. Also, the Bocas del Toro specimens (Litvaitis 2014–2024) show specimens with and without pigmentation.

Although some doubts have arisen about the identity of this species, the presence or absence of pigmentation spots in the preserved specimens of *Idioplana* described from the Gulf of Mexico does not provide sufficient evidence to confirm the existence of a new species. Therefore, we have decided to classify the *Idioplana* specimens found in Yucatan as *I.atlantica*. Further morphological and molecular studies will be necessary to determine whether it represents a distinct species.

### ﻿Stylochidae Stimpson, 1857

***Stylochus* Ehrenberg, 1831**

#### ﻿ Stylochus sixteni

Taxon classificationAnimalia﻿Polycladida﻿Stylochidae

Marcus, 1947

B4FEFFA4-5553-582F-915A-3FE1AC16E0CE

Fig. 24

##### Material examined.

**Yucatan coast, Mexico** • 1; Dzilam; 21.5°N, 88.9°W; 9.3 m; 8 May 2018; A. Hernández leg.; CRPPY-0090 • 1; 12 slides; Dzilam; 21.5°N, 88.9°W; 13 m; 10 May 2018; A. Hernández leg.; CRPPY-0099.

##### Distribution.

The species was originally described in Cape Verde (Bock 1931). New record for the Gulf of Mexico (Yucatan).

##### Description.

**Figure 24.**
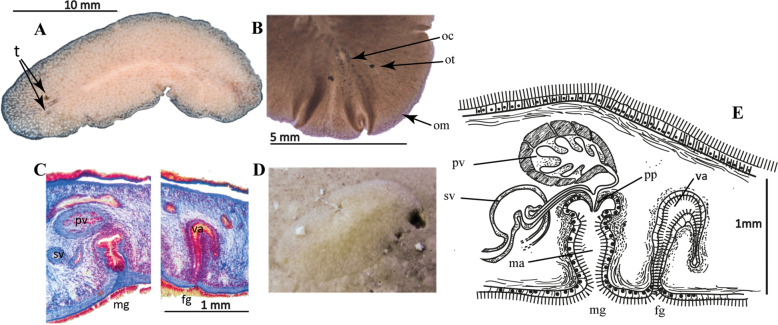
*Stylochussixteni***A** live animal, photographed on a black background, tentacles **B** anterior end, marginal, cerebral and tentacular eyes, after fixation **C** sagittal section through the reproductive system (stained with AZAN) **D** specimen in situ **E** sagittal reconstruction of male and female copulatory organs.

Elongated oval body shape, firm and fleshy consistency. In live specimens, body measures 20 mm in length, and 7 mm in width, whereas in fixed specimens, measures are reduced to 10 mm length, and 8 mm in width. Rounded nuchal tentacles visible. Pale beige colouration, translucent near the margins. Small white spots, denser along the midline and a white dotted line along the entire body margin (Fig. 24A, D). Two elongated clusters of cerebral eyes between nuchal tentacles. Densely packed tentacular eyes. Marginal eyes in the first 1/3 of the body, up to the level of the tentacles (Fig. 24B). Ruffled pharynx centrally located, oral opening at its centre. ***Reproductive system*.** Male and female copulatory apparatus posterior to pharynx, near second 1/2 of body. Male apparatus comprises a free prostatic vesicle and a small kidney-shaped seminal vesicle. Ejaculatory duct penetrates through an anteroposterior-oriented penis papilla into the deep atrium. Penis papilla, small, conical. Male atrium heart-shaped, deep (Fig. 24C, E). Female apparatus simple, anteroposteriorly oriented, slightly muscular vagina, and small ciliated female atrium (Fig. 24C, E).

##### Remarks.

To avoid confusion between *Stylochuscrassus* Verrill, 1893 (from the coast of Maryland, USA) and *S.crassus* Bock, 1931 (from the coast of Cape Verde Island), Marcus (1947) renamed the specimens described by Bock (1931) as *Stylochussixteni*. The specimen captured on the coast of Yucatan is most similar to the specimen described by Bock (1931). The major difference between the specimens described by Bock (1931) and *Stylochussixteni* from the Gulf of Mexico is the distance of the reproductive system from the posterior end of the animal. The reproductive organs, in Bock’s description, are close to the body end, while in the Yucatan specimens, they are near to the posterior end, but not so close as in Bock’s. However, this may be due to the difference in size, since the specimens from Cape Verde measured 10 mm, while those from the Yucatan coast measured 20 mm (Fig. 24E).

### ﻿Hoploplanidae Stummer-Traunfels, 1933

***Hoploplana* Laidlaw 1902**

#### ﻿ Hoploplana inquilina

Taxon classificationAnimalia﻿Polycladida﻿Hoploplanidae

(Wheeler, 1894)

EDF550A3-36BB-54EC-A08A-4AABEB46FC86

Fig. 25A–D

##### Material examined.

**Yucatan coast, Mexico** • 1; Dzilam; 21.5°N, 88.9°W; 9.3 m; 8 May 2018; A. Hernández leg.; CRPPY-0089.

##### Distribution.

*Hoploplanainquilina* has been observed off St. Thomas Island, Caribbean (Hyman 1939). It has been reported in the Gulf of Mexico, Bermuda, and the Central North Atlantic (Prudhoe 1985); Cayman Islands, Caribbean (Hyman 1954). Additionally, it has been found in shells in Massachusetts (Hyman 1939, 1940). Specimens have also been documented in the mantle-cavity of *Urosalpinxcinerea* and *Eupleuracaudata* in New Jersey (Schlechter 1943), and in the mantle-cavity of *Thaisfloridana* in Florida and Louisiana (Hyman 1944, 1954).

##### Description.

**Figure 25.**
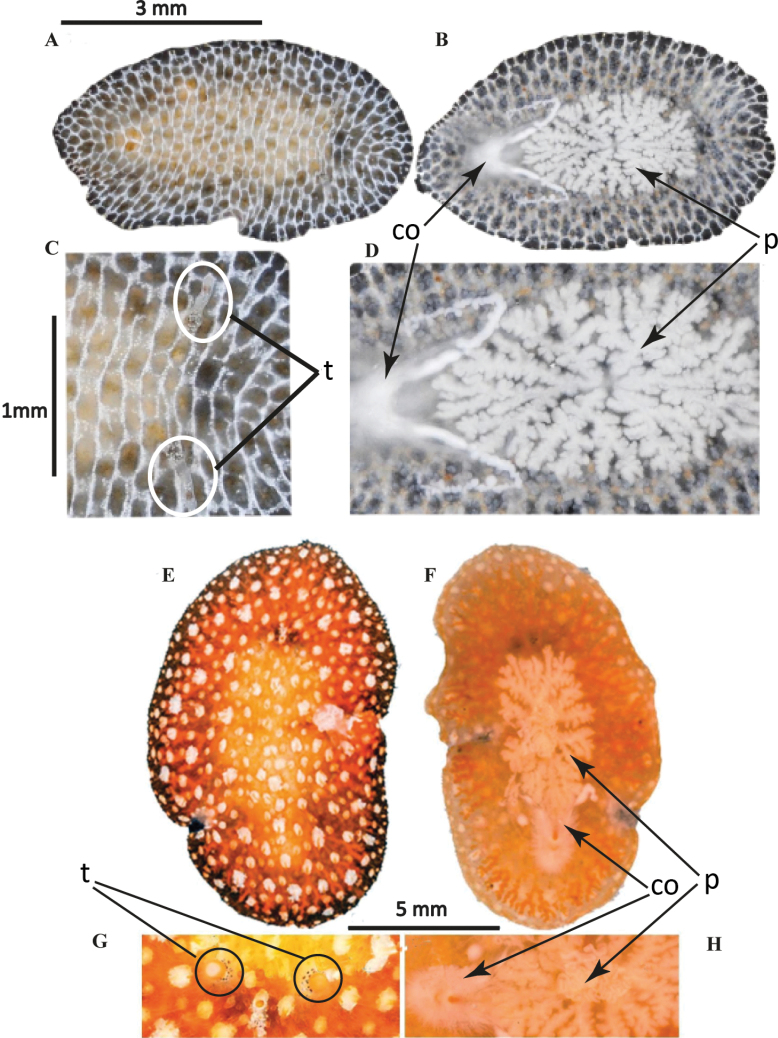
**A–D***Hoploplanainquilina***A** dorsal view **B** ventral view **C** anterior end, tentacles and tentacular eyes **D** ventral view, ruffled pharynx and reproduction organs **E–H***Hoploplanadivae***E** dorsal view **F** ventral view **G** anterior end, tentacles **H** ventral view, ruffled pharynx and reproduction organ.

Body shape oval, 5 mm in length and 3 mm in width. Translucent bluish-to-grey background colour with a distinctive white reticulum on the dorsal surface that does not correspond to the intestine (Fig. 25A, B). This network extends to the periphery, appearing white in reflected light and black in transmitted light, as described by Wheeler (1894). Delicate tubular nuchal tentacles present. Two clusters of small, rounded cerebral eyes situated between the nuchal tentacles, with tentacular eyes at the base (Fig. 25C). Ruffled pharynx, branched intestine, and reproductive system milky white and visible dorsally and ventrally (Fig. 25B, D).

#### ﻿ Hoploplana divae

Taxon classificationAnimalia﻿Polycladida﻿Hoploplanidae

Marcus, 1950

A653467A-83B3-5E00-BD23-B801138A4B99

Fig. 25E–H

##### Material examined.

**Campeche coast, Mexico** • 1; Cayo Arcas; 20.2°N, 92.0°W; 5.3 m; 23 Apr. 2018; A. Hernández leg.; CRPPY-0073 • 1; Cayo Arcas; 20.2°N, 92.0°W; 13.2 m; 24 Apr. 2018; A. Hernández leg.; CRPPY-0080.

##### Distribution.

Originally described in São Paulo, Southeast of Brazil (Marcus 1950) and later by Bahia and Schrödl (2018). *Holoplanadivae* has been documented in Rio Grande do Norte, Brazil (Bahia et al. 2012) and Curaçao (Marcus and Marcus 1968). This study marks a novel record for the Campeche coast and new record for the Gulf of Mexico.

##### Description.

Body shape oval with rounded anterior and posterior end, 1.2 cm in length and 0.6 cm in width. Two small cylindrical nuchal tentacles (Fig. 25E, G). Tentacular eyes at base of tentacles. Cerebral eyes two sparse clusters, extending towards anterior and posterior regions of body. Colouration semi-translucent, orange to pinkish. Dorsal epidermis covered with numerous semi-cylindrical whitish papillae. Largest papillae in posterior region. Highly folded ruffled pharynx, characteristic of the species of the genus *Hoploplana*. Oral opening situated in the anterior 1/2 of the body (Fig. 25F, H). Male and female gonopores close to each other, distinctly separated and open near the posterior end (Fig. 25F, H).

## ﻿Conclusions

This study provides a valuable contribution to our knowledge of Polycladida diversity in the southern regions of the Gulf of Mexico. Our research reveals the presence of 27 polyclad species, belonging to 17 genera and 12 families. By revising the polyclad records in the Gulf of Mexico, we have increased the known species count from 31 to 50. It is noteworthy that this is the first known report of marine flatworms along the coasts of Campeche, Yucatan, and Quintana Roo.

This study has identified 17 species that were previously unknown in the Gulf of Mexico, thus expanding their known distribution ranges. Some of the notable findings include the extension of distribution ranges for *Enchiridiumevelinae*, *Pseudocerosjuani*, *Phaenocelispeleca*, *Stylochussixteni*, and *Hoploplanadivae*. Additionally, this study has introduced two new species, *Stylochoplanasisalensis* sp. nov. and *Emprosthopharynxhartei* sp. nov. The latter marks the first report of its genus on the Atlantic coast of the Americas.

This work highlights the rich diversity of Polycladida along the Atlantic coastline of Mexico. It also emphasises the importance of exploring and documenting under-researched species, particularly in regions home to abundant fauna. Ultimately, our study contributes to the development of a comprehensive atlas of unrecorded species, which will help to enhance conservation efforts and advance our knowledge of marine biodiversity in the Gulf of Mexico.

## Supplementary Material

XML Treatment for Pericelis cata

XML Treatment for Pericelis orbicularia

XML Treatment for Prosthiostomum utarum

XML Treatment for Enchiridium evelinae

XML Treatment for Enchiridium periommatum

XML Treatment for Eurylepta aurantiaca

XML Treatment for Prostheceraeus crozieri

XML Treatment for Pseudoceros bicolor

XML Treatment for Pseudoceros rawlinsonae

XML Treatment for Pseudoceros bolool

XML Treatment for Pseudoceros juani

XML Treatment for Pseudobiceros caribbensis

XML Treatment for Pseudobiceros splendidus

XML Treatment for Pseudobiceros pardalis

XML Treatment for Phrikoceros mopsus

XML Treatment for Thysanozoon brocchii

XML Treatment for Phaenocelis medvedica

XML Treatment for Phaenocelis peleca

XML Treatment for Stylochoplana sisalensis

XML Treatment for Emprosthopharynx hartei

XML Treatment for Notoplana annula

XML Treatment for Notocomplana ferruginea

XML Treatment for Gnesioceros sargassicola

XML Treatment for Idioplana atlantica

XML Treatment for Stylochus sixteni

XML Treatment for Hoploplana inquilina

XML Treatment for Hoploplana divae

## ﻿Additional information

### Author ORCIDs

Daniel Cuadrado https://orcid.org/0000-0001-5565-3023

Alejandro Hernández-González https://orcid.org/0000-0002-1557-5582

Carolina Noreña https://orcid.org/0000-0002-4580-7460

Nuno Simões https://orcid.org/0000-0001-7490-3147

### Sampling and field studies

The samples were collected under the collection permit N. PPF / DGOPA-295/17 and PFF / DGOPA-079/19, issued by Mexico’s State Secretariat of Agriculture, Livestock, Rural Development, Fisheries, and Food (SAGARPA). Access to Alacranes Reef National Park was authorised by the Park authorities and we appreciate the possibility of using their facilities in Perez Island.

### Data availability

Whole body specimens and the histological preparations of those specimens under study were deposited at the 'Colección regional de Policladidos de la Península del Yucatán, Mexico' (CRPPY), located at the Multidisciplinary Teaching and Research Unit of Sisal, Faculty of Sciences, National Autonomous University of Mexico (UMDI-Sisal, FC-UNAM). All sequences obtained in the present study have been deposited in the GenBank (Suppl. material 1: table S2).

## Supplementary materials

Supplementary material 1 Supplementary information https://doi.org/10.3897/zookeys.1221.128260.suppl1 Data type docx Explanation note **table S1.**Polycladida of the Gulf of Mexico, updated and with species studied, new records for the Gulf of Mexico and new species added. **table S2.** GenBank accession numbers of the molecular sequences generated in this study of 28S from Polycladida of the Gulf of Mexico. https://binary.pensoft.net/file/1197998This dataset is made available under the Open Database License (http://opendatacommons.org/licenses/odbl/1.0/). The Open Database License (ODbL) is a license agreement intended to allow users to freely share, modify, and use this Dataset while maintaining this same freedom for others, provided that the original source and author(s) are credited.Daniel Cuadrado, Alejandro Hernández-González, Carolina Noreña, Nuno Simões
