# Supplementary material for: ﻿Polyclads (Platyhelminthes) in the southern Gulf of Mexico: unveiling biodiversity and descriptions of two new species
Source: Zookeys. 2024 Dec 13;1221:103–44. doi: 10.3897/zookeys.1221.128260 (PMC11662208; doi:10.3897/zookeys.1221.128260)
Supplement: Supplementary material 1 — Supplementary information [file zookeys-1221-103_article-128260__-s001.docx]

**Table S1**: Polycladida of the Gulf of Mexico, updated and with species studied (*), new records for the Gulf of Mexico (**) and new species (***) added. Abbreviations: ben = benthic; bns = bay and nearshore; bsl = beach and shoreline; cep = coastal surface and epipelagic; epi = epibiotic; hsb = hard substrate; plk = planktonic; sym = symbiotic; - = without data.

|  | Order Polycladida  Suborder: Acotylea | Habitat | Depth (meters) | Geographic range | References |
| --- | --- | --- | --- | --- | --- |
| 1 | *Stylochoplana sisalensis* sp. nov. | ben | 0-2 | Gulf of Mexico (Campeche) | *** |
| 2 | *Phaenocelis purpurea* (Schmarda, 1859) | ben, bsl, hsb | 0–1 | Gulf of Mexico (Florida)  Caribbean Sea (Curaçao) | Hyman (1944, 1954, 1955, 1955),  Marcus and Marcus (1968) |
| 3 | *Phaenocelis medvedica* Marcus, 1952 | ben | - | SW Atlantic (Brazil)  Caribbean Sea (Colombia)  Gulf of Mexico (Campeche) | Marcus (1952),  Bahia et al. (2015), Bahia and Schrödl (2018),  Quiroga et al. (2004a)  ** |
| 4 | *Phaenocelis peleca* Marcus & Marcus, 1968 | ben | 0-2 | Caribbean Sea (Curaçao)  Gulf de Mexico (Campeche) | Marcus and Marcus (1968)  ** |
| 5 | *Coronadena mutabilis* (Verrill, 1873) | ben, plg | 0–2 | NW Atlantic (Massachusetts)  Gulf of Mexico (Florida) | Pearse (1938),  Hyman (1954) |
| 6 | *Spinantia pellucida* (Pearse, 1938) | ben, bsl | 0–3 | Gulf of Mexico (Florida) | Pearse (1938) |
| 7 | *Gnesioceros floridana* (Pearse, 1938) | ben, bsl | - | Gulf of Mexico (Texas to Florida)  NW Atlantic (Massachusetts, Virgin Is.) | Hyman (1939, 1954, 1955) |
| 8 | *Gnesioceros sargassicola* (Mertens, 1833) | epi, cep | 0 | Gulf of Mexico (Texas to Florida)  NW Atlantic (Bemudas, Sargasso Sea)  Caribbean Sea (Colombia,  Curaçao, Netherlands Antilles)  Gulf of Mexico (Yucatan) | Hyman (1939, 1954),  Quiroga (2008),  Marcus and Marcus (1968)  * |
| 9 | *Chatziplana grubei*  (Graff, 1892) | plk, cep, epi | 0 | NW Atlantic (Massachusetts, Sargasso Sea)  Gulf of Mexico (Texas to Florida) | Hyman (1939, 1954) |
| 10 | *Hoploplana inquilina* (Wheeler, 1894) | ben, bsl, sym | 0–2 | Gulf of Mexico (Florida, Louisiana, Texas)  NW Atlantic (Massachusetts)  Gulf of Mexico (Yucatan) | Hyman (1039, 1940, 1944, 1954), Prudhoe (1985), Schechter (1943)  * |
| 11 | *Hoploplana divae* Marcus, 1950 | ben | - | SW Atlantic (Brazil)  Caribbean Sea (Curaçao)  Gulf of Mexico (Campeche) | Bahia et al. (2012), Bahia and Schrödl (2018), Marcus (1950)  Marcus and Marcus (1968)  ** |
| 12 | *Latocestus whartoni* (Pearse, 1938) | ben, bsl | 0–3 | Gulf of Mexico (Florida),  NW Atlantic (North Carolina) | Pearse (1938),  Hyman (1940, 1954) |
| 13 | *Didangia carneyi* Quiroga, Bolaños & Litvaitis, 2008 |  | 610 | Gulf of Mexico (Louisiana) | Quiroga (2008) |
| 14 | *Digynopora americana* Hyman,1940 | bns, hsb | 0–2 | Gulf of Mexico (Florida) | Hyman (1940) |
| 15 | *Euplana gracilis* Girard, 1850 | ben, bsl | 0–3 | NW, NE Atlantic  Gulf of Mexico (Florida) | Hyman (1939, 1940, 1954),  Prudhoe (1982), Pearse (1938) |
| 16 | *Notoplana* *annula* Marcus & Marcus, 1968 | ben | 0-2 | Caribbean Sea (Curaçao)  NW Atlantic (Florida)  Gulf of Mexico (Campeche) | Marcus and Marcus (1968)  ** |
| 17 | *Comoplana angusta* (Verrill, 1893) | bsl, hsb | 0–3 | Gulf of Mexico (Florida)  NW, SW, SE Atlantic | Hyman (1940, 1954),  Verrill (1893), Palombi (1928), Marcus (1947) |
| 18 | *Stylochus oculifera* Girard, 1853 | ben, bsl | 2–3 | Gulf of Mexico (Florida)  NW Atlantic  Caribbean Sea (Curaçao) | Pearse (1938),  Hyman (1940, 1954),  Marcus and Marcus (1968) |
| 19 | *Stylochus ellipticus* (Girard, 1850) | ben, bsl | 0–3 | Gulf of Mexico (Texas to Florida)  NW Atlantic | Hyman (1940, 1954),  Pearse (1938) |
| 20 | *Stylochus frontalis* Verrill, 1892 | ben, bsl | 2–3 | Gulf of Mexico (Texas to Florida)  Caribbean Sea (Curaçao) | Hyman (1954)  Marcus and Marcus (1968) |
| 21 | *Stylochus sixteni* Marcus, 1947 | ben | 3 | Cape Verde  Gulf of Mexico (Yucatan) | Bock (1931)  ** |
| 22 | *Idioplana atlantica*Bock, 1913 | ben | 3 | Caribbean Sea (Cuba, Virgin Island USA, Panama)  Gulf of México (Yucatan) | Catalá et al. (2016), Bock (1913), Rawlinson, (2008), Quiroga et al. (2004b)  ** |
| 23 | *Notocomplana ferruginea* (Schmarda, 1859) | ben | 0-3 | Caribbean Sea (Colombia, Puerto Rico, Curaçao, Jamaica)  Gulf of Mexico (Veracruz) | Marcus and Marcus (1968), Quiroga et al. (2004a), Hyman (1955)  ** |
| 24 | *Notocomplana lapunda* (Marcus & Marcus, 1968) |  |  | NW Atlantic (Florida) Caribbean Sea (Curaçao, Panama) | Marcus and Marcus (1968),  Litvaitis et al. (2019) |
| 25 | *Emprosthopharynx hartei* sp. nov. | ben | 14 | Gulf of Mexico (Yucatan) | *** |
|  | **Suborden Cotylea** |  |  |  |  |
| 26 | *Eurylepta aurantiaca* Heath & McGregor, 1912 | ben | 12 | NE Pacific (Canada to California)  SW Atlantic (Brazil)  Caribbean Sea (Colombia)  India  Gulf of Mexico (Yucatan) | Hyman (1953, 1955)  Bahia et al. (2014),  Quiroga et al. (2004a, 2004b),  Pitale and Apte (2019)  ** |
| 27 | *Eurylepta multicelis* (Hyman, 1955) | - | des | Gulf of Mexico (Florida)  NW Atlantic (Florida, Puerto Rico) | Hyman (1955) |
| 28 | *Acerotisa baiae* Hyman, 1940 | - | des | Gulf of Mexico (Florida)  NW Atlantic (Massachusetts) | Hyman (1940),  Hyman (1952) |
| 29 | *Oligocladus floridanus* Pearse, 1938 | ben, bsl | 0–2 | Gulf of Mexico (Florida),  NW Atlantic (North Carolina) | Pearse (1938),  Hyman (1940, 1954) |
| 30 | *Oligocladus bathymodiensis* Quiroga, Bolaños & Litvaitis, 2008 |  | 650 | Gulf of Mexico (Louisiana) | Quiroga et al. (2008) |
| 31 | *Prostheceraeus crozieri* (Hyman, 1939) | ben, bsl, bns, epi | 0–3 | NW Atlantic (Virgin Is.),  Caribbean Sea (Belize, Honduras, Curaçao)  Gulf of Mexico (Campeche, Yucatan) | Marcus and Marcus (1968),  Hyman (1939, 1952), Crozier (1917)  ** |
| 32 | *Prostheceraeus floridanus* Hyman, 1955 | ben, end | des | Gulf of Mexico (Florida)  NW Atlantic (Virgin Is.)  Caribbean Sea (Belize, Honduras) | Hyman (1955b)  Bolanos et al. (2007) |
| 33 | *Pericelis orbicularia* (Schmarda, 1859) | ben, bsl | 0-3 | Gulf of Mexico (Texas to Florida),  Caribbean Sea (Jamaica)  Gulf of Mexico (Yucatan) | Hyman (1955),  Marcus and Marcus (1968)  Schmarda (1859)  * |
| 34 | *Pericelis cata* Marcus & Marcus, 1968 | ben | 0–2 | Caribbean Sea (Belize, Curaçao, Colombia, Jamaica, Virgin Is.)  SW Atlantic (Brazil)  Spain (Canary Islands)  Gulf of Mexico (Campeche)  Mexican Caribbean Sea (Quintana Roo) | Quiroga et al. (2004a),  Rawlinson (2008),  Queiroz et al. (2013),  Bahia et al. (2014, 2015), Bahia and Schrödl (2018),  Cuadrado et al. (2017)  **  * |
| 35 | *Enchiridium periommatum* Bock, 1913 | ben, bsl | 12 | Gulf of Mexico (Texas to Florida)  Caribbean Sea (Jamaica)  Gulf of Mexico (Campeche)  Mexican Caribbean Sea (Quintana Roo) | Hyman (1955),  Bock (1913)  *  * |
| 36 | *Prosthiostomum lobatum* Pearse, 1938 | ben, bsl | 0–3 | Gulf of Mexico (Florida),  NW Atlantic (North Carolina)  Caribbean Sea (Jamaica) | Pearse (1938),  Hyman (1940, 1954),  Rawlinson (2008) |
| 37 | *Prosthiostomum utarum* Marcus, 1952 | ben | 3 | SW-Atlantic (Brazil)  Caribbean Sea (Jamaica, Colombia, Curaçao, Virgin Is.)  Mexican Caribbean Sea (Quintana Roo) | Bahia et al. (2014),  Bahia and Schrödl (2018), Quiroga et al. (2004a)  * |
| 38 | *Enchiridium evelinae* Marcus, 1949 | ben | 3 | SW Atlantic (Brazil)  Caribbean Sea (Curaçao)  Panama  Gulf of Mexico (Yucatan) | Marcus (1947)  Bahia et al. (2012, 2014, 2015), Bahia and Schrödl (2018),  Marcus and Marcus (1968),  Rawlinson (2008)  ** |
| 39 | *Acanthozoon maculosum*(Pearse, 1938) | ben | des | Gulf of Mexico (Florida) | Pearse (1938),  Hyman (1954) |
| 40 | *Thysanozoon brocchi* (Risso, 1818) | ben, bsl | 0–2 | Cosmopolitan  Caribbean Sea (Curaçao)  SW Atlantic (Brazil)  Argentina  Gulf of Mexico (Florida, Campeche) | Prudhoe (1985), Hyman (1952),  Bahia et al. (2014, 2015, 2017), Bahia and Schrödl (2018),  Brusa et al. (2009),  Marcus and Marcus (1968)  * |
| 41 | *Thysanozoon nigrum* (Girard, 1852) | ben, bsl | 0–2 | NW Atlantic (Bermuda, Bahamas)  Gulf of Mexico (Texas, Florida) | Hyman (1939),  Hyman (1952, 1955),  Marcus and Marcus (1968) |
| 42 | *Pseudoceros (?) texanus* Hyman, 1955 | bns, epi | 0–2 | Gulf of Mexico (Texas)  Caribbean Sea (Antilles) | Hyman (1955b),  Marcus and Marcus (1968) |
| 43 | *Pseudoceros juani* Bahia, Padula, Lavrado & Quiroga, 2014 | ben | 5 | Brazil (Cabo Frío)  Gulf of Mexico (Campeche) | Bahia et al. (2014)  ** |
| 44 | *Pseudoceros bicolor* Verrill, 1902 | ben | 3–11 | Gulf of Mexico (Florida)  Caribbean Sea (Jamaica, Belize, Curaçao, Honduras, Panama, Virgin Is.)  SW Atlantic (Brazil)  Gulf of Mexico (Campeche, Yucatan)  Mexican Caribbean Sea (Quintana Roo) | Rawlinson (2008),  Litvaitis et al. (2010),  Marcus and Marcus (1968), Quiroga et al. (2004a),  Bahia and Padula (2009), Bahia et al. (2014, 2015), Bahia and Schrödl (2018)  *  * |
| 45 | *Pseudoceros bolool* Newman *&* Cannon*,* 1994 | ben | 22 | NW Atlantic (Florida)  India  Pacific Ocean (Australia)  Gulf of Mexico (Campeche) | Rawlinson (2008),  Thakkar et al. (2017), Sreeraj et al. (2015),  Newmann and Cannon (1994)  ** |
| 46 | *Pseudoceros rawlinsonae* Bolaños*,* Quiroga & Litvaitis, 2007 | ben | 10 | NW Atlantic (Florida)  Caribean Sea (Jamaica, Bahamas, Honduras, Virgin Is., Curaçao)  SW Atlantic (Brazil)  Gulf of Mexico (Campeche, Yucatan)  Mexican Caribbean Sea (Quintana Roo) | Bolaños et al. (2007),  Litvaitis et al. (2010, 2019),  Bahia et al. (2014, 2015), Bahia and Schrödl (2018)  **  * |
| 47 | *Phrikoceros mopsus* (Marcus, 1952) | ben | 13 | Caribbean Sea (Curaçao)  SW Atlantic (Brazil, Argentina)  India  Gulf of Mexico (Campeche)  Mexican Caribbean Sea (Quintana Roo) | Marcus and Marcus (1968),  Bahia et al. (2012, 2014, 2015, 2017), Bahia and Schrödl (2018), Brusa et al. (2009),  Sreeraj and Raghunathan (2015)  **  * |
| 48 | *Pseudobiceros caribbensis* Bolaños*,* Quiroga & Litvaitis, 2007 | ben | 8–13 | NW-Atlantic (Florida)  Caribbean Sea (Curaçao, Jamaica, Honduras)  Gulf of Mexico (Campeche) | Bolaños et al. (2007),  Rawlinson (2008)  ** |
| 49 | *Pseudobiceros splendidus* (Lang, 1884) Marcus, 1950 | ben | 7 | NW Atlantic (Bermuda, Florida)  Ecuador (Galapagos Is.)  Puerto Rico  Brazil  Australia  New Guinea  Gulf of Mexico (Yucatan) | Verrill (1900), Hyman (1939, 1955), Marcus (1950), Plehn (1896)  Bahia et al. (2012, 2014), Bahia and Schrödl (2018),  Newman and Cannon (1994, 1997), Marquina et al. (2015)  ** |
| 50 | *Pseudobiceros pardalis* (Verrill 1900) | ben | des | Caribbean Sea (Panama)  NW Atlantic (Bermuda, Florida, Bahamas)  SW Atlantic (Brazil)  Mexican Caribbean Sea (Quintana Roo) | Bolaños et al. (2007),  Marcus (1950)  Bahia et al. (2012, 2014, 2015)  * |

**Table S2**: GenBank accession numbers of the molecular sequences generated in this study of 28S from Polycladida of the Gulf of Mexico.

| Family | Species | 28S |
| --- | --- | --- |
| Pericelidae Laidlaw, 1902 | *Pericelis orbicularia* | MT677885 |
| Euryleptidae Stimpson, 1857 | *Prostheceraeus crozieri* | MT677881 |
| Pseudocerotidae Faubel, 1984 | *Pseudoceros bicolor* | MT677879 |
|  |  | MT677880 |
|  | *Pseudobiceros caribbensis* | MT677878 |
| Cryptocelididae Laidlaw, 1903 | *Phaenocelis medvedica* | MT677877 |
| Stylochoplanidae Faubel, 1983 | *Emprosthopharynx hartei* sp. nov. | MT677882 |
|  |  | MT677883 |
| Notocomplanidae Litvaitis, Bolaños & Quiroga, 2019 | *Notoplana ferruginea* | MT677887 |
| Idioplanidae Dittmann, Cuadrado, Aguado, Noreña & Egger, 2019 | *Idioplana atlantica* | MT677884 |
| Stylochidae Stimpson, 1857 | *Stylochus sixteni* | MT677886 |
